# Serum PFAS concentrations and neuromorphometry in adolescents: The HOME Study

**DOI:** 10.1016/j.envres.2026.124338

**Published:** 2026-03-20

**Authors:** Jasmine Haraburda, Jonathan Dudley, Kimberly Yolton, Yingying Xu, Joseph M. Braun, Aimin Chen, Bruce P. Lanphear, Kim M. Cecil

**Affiliations:** a University of Cincinnati College of Medicine, Cincinnati, OH, USA; b Imaging Research Center, Cincinnati Children’s Hospital Medical Center, Cincinnati, OH, USA; c Department of Pediatrics, Cincinnati Children’s Hospital Medical Center, University of Cincinnati College of Medicine, Cincinnati, OH, USA; d Department of Environmental and Public Health Sciences, University of Cincinnati College of Medicine, Cincinnati, OH, USA; e Department of Epidemiology, Brown University School of Public Health, Providence, RI, USA; f Department of Biostatistics, Epidemiology and Informatics, University of Pennsylvania Perelman School of Medicine, Philadelphia, PA, USA; g Department of Health Sciences, Simon Fraser University, Burnaby, BC, Canada; h Department of Radiology, Cincinnati Children’s Hospital Medical Center, University of Cincinnati College of Medicine, Cincinnati, OH, USA

**Keywords:** Per- and polyfluoroalkyl substances, Magnetic resonance imaging, Neuromorphometry, Adolescent

## Abstract

**Background:**

Per- and polyfluoroalkyl substances (PFAS) are a ubiquitous class of persistent synthetic chemicals used in consumer and commercial products for decades. PFAS crosses the blood-placenta and blood-brain barriers to accumulate in the fetus, but the impact on neuroanatomy in early adolescence is unknown.

**Objectives:**

We estimated the association of gestational and early adolescent PFAS concentrations with brain morphometry in 155 participants of the Health Outcomes and Measures of the Environment (HOME) Study, a prospective pregnancy and birth cohort enrolled from 2003 to 2006.

**Methods:**

We measured serum PFAS concentrations during gestation and at age 12. We acquired high resolution anatomical brain MRI to quantify global and regional volumes, cortical thickness and sulcal depth using morphometric methods at age 12. We used a general linear model framework with adjustment for adolescent biological sex, race, household income, maternal IQ, maternal pre-pregnancy BMI, primipara, and total intracranial volume.

**Results:**

A doubling in age 12 perfluorooctanoic acid (PFOA) [f^2^ = 0.06, β = 10.79, (95% CI: 3.45, 18.13), p = 0.04] and perfluorononanoic acid (PFNA) [f^2^ = 0.04, β = 6.14, (95%CI: 1.09, 11.18), p = 0.018] concentrations were positively associated with total gray matter volume. A doubling in age 12 PFOA [f^2^ = 0.04, β = −8.49, (95% CI: −15.28, −1.69), p = 0.015] and perfluorooctanesulfonic acid (PFOS) [f^2^ = 0.03, β = −5.00, (95% CI: −9.81, −0.18), p = 0.042] concentrations were negatively associated with total white matter volume. A doubling in age 12 PFOA [(f^2^ = 0.03, β = 0.03, (95% CI: 0.00, 0.06), p = 0.034] and PFOS [(f^2^ = 0.03, β = 0.02, (95% CI: 0.00, 0.04), p = 0.035] concentrations were positively associated with whole brain average cortical thickness. A doubling in age 12 PFOA was positively associated with regional gray matter volumes up to 0.05 mm^3^ within cortical, subcortical and deep gray matter, and cortical thickness up to 0.1 mm predominately within the posterior cortex, respectively. No significant associations were observed for gestational PFAS concentrations and brain morphometry.

**Conclusion:**

Higher PFAS concentrations are concurrently associated with larger structural volumes and cortical thickness. The significance is uncertain but are consistent with delayed maturation processes delaying synaptic pruning.

## 1. Introduction

Per- and polyfluoroalkyl substances (PFAS) are a large group of synthetic chemicals generally characterized as partially or fully fluorinated aliphatic compounds with amphiphilic properties (Buck et al., 2011). PFAS have been widely utilized in the production of numerous industrial and consumer products including surfactants, food packaging, fire-fighting foams, oil and water repellent textiles, non-stick cookware, waxes, personal care products (e.g. dental floss, cosmetics, sunscreens), furniture, and pesticides since the 1940s (ATSDR, 2021; Buck et al., 2011). PFAS are referred to as “forever chemicals” due to persistence imparted by strong carbon-fluorine bonds (Kwiatkowski et al., 2020) making products thermally and chemically stable (Glüge et al., 2020). Because of their pervasiveness and persistence, PFAS significantly impact both the natural environment with contamination of soil, sediment and groundwater, and human health with liver damage, thyroid disease, immune effects, increases in serum lipids, reproductive and developmental toxicity (ATSDR, 2021).

Workers are exposed to PFAS through occupational sources throughout fluoropolymer manufacturing processes and product usage. Ubiquitous human PFAS exposure occurs with activities of daily living including the use of consumer products and food consumption. Airborne particulates with PFAS are emitted from manufacturing facilities, landfills and firefighting foam. PFAS contamination of drinking water and food, especially seafood, occurs from contamination of surface soils, groundwater and rivers, with additional exposures from food packaging, storage supplies and cookware. The pathways of exposure occur via oral ingestion, inhalation, and dermal contact (Zhang et al., 2022). PFAS are measurable in biological matrices due to their high affinity for proteins (De Silva et al., 2021; Domingo, 2025; Gyllenhammar et al., 2018; VanNoy et al., 2018). PFAS exhibit varied accumulation patterns based on tissue type (De Silva et al., 2021.) Due to their small size and chemical properties, PFAS can cross the blood-brain-barrier (BBB) and the placenta (Appel et al., 2022; Bharal et al., 2024; Ma et al., 2022; Midasch et al., 2007; Starnes et al., 2022; Wang et al., 2018). While PFAS predominately accumulates in the liver and blood (Domingo, 2025; Jia et al., 2022), the brain is especially prone to bioaccumulation of PFAS given its high degree of vascularization (Starnes et al., 2022). PFAS has also been shown to amass in the brain with the brainstem, hippocampus, hypothalamus, pons, medulla, and thalamus as the main sites of accumulation in polar bears (Cao and Ng, 2021; Eggers Pedersen et al., 2015; Greaves et al., 2013). In brain autopsy studies from persons who lived in a region with high PFAS contamination, Di Nisio et al. reported high levels of accumulation in areas of the basal ganglia such as the caudate nucleus, lenticular nucleus and especially the hypothalamus (Di Nisio et al., 2022).

Exposure to and/or accumulation of PFAS in the brain may impact neurodevelopment as some neurobehavioral studies link early life PFAS exposure to reduced cognitive, language and motor development in infancy and increased attention problems, hyperactivity and reduced ability to perform activities of daily living in childhood (Ames et al., 2025; Lagostena et al., 2025; Vuong et al., 2021b). There are currently only three neuroimaging studies published evaluating the effects of gestational PFAS on children. Barron et al. (2025) evaluated 51 mother-child dyads of the FinnBrain Birth Cohort to examine the association of maternal PFAS concentrations at 24 weeks gestation and multiple structural and functional brain MRI-based features simultaneously with independent component analysis in children at age 5 years. Employing multiple robust statistical techniques, maternal PFNA and linear PFOA linearly predicted a multimodal component from diffusion imaging with diminished integrity of the corpus callosum, a key white matter track. They also found that branched PFOA and branched perfluorohexanesulfonic acid (PFHxS) predicted a component including occipital cortex volume and surface area. In a sub-cohort of the Canadian Alberta Pregnancy Outcomes and Nutrition Study, England-Mason et al. reported higher second trimester maternal PFAS plasma concentrations were associated with altered white matter microstructure evaluated from 2 to 6 years of age with longitudinal diffusion tensor imaging (England-Mason et al., 2025). In the body and splenium of the corpus callosum, maternal perfluoroalkyl sulfonates were associated with tract-based diffusion metrics indicating lower fractional anisotropy and higher mean diffusivity values. The third study evaluated prenatal PFAS and phthalate concentrations with morphometry and diffusion tensor imaging outcomes in 52 adolescents (ages 14–15 years) from Taiwan. Wang et al. showed significant associations between prenatal exposure to PFAS and phthalates with changes in specific fronto-parietal regions of the adolescent male brain, including reduced cortical thickness in the inferior frontal gyrus and right superior parietal cortex (Wang et al., 2025). Our objective was to estimate the associations of gestational and early adolescent serum PFAS concentrations with morphometric features obtained from anatomical brain MRI acquired at the age 12 study visit from participants of the Health Outcomes and Measures of the Environment (HOME) Study. As noted by our own neurobehavioral and from the three neuroimaging studies, the prenatal PFAS exposure timepoint is a critical window (Vuong et al., 2021a, 2021b). Also, the changes in brain plasticity, dynamic psychosocial changes impacting behavior and the pubertal maturation of all organ systems makes early adolescence a second sensitive developmental period (Viner et al., 2015). We selected morphometry as our focused neuroimaging outcome as it provides sensitivity to directly interpretable structural measurements such as volume and cortical thickness without complex modeling required in diffusion imaging to accurately characterize changes. Morphometry holds high consistency and reliability across imaging sites with less artifacts compared to diffusion imaging. The HOME Study provided a larger sample size for the analyses compared to the existing studies in the literature. To our knowledge, this will be the first study reporting the relationship of early adolescent PFAS concentrations and concurrent brain morphometry.

## 2. Methods

### 2.1. Study participants

The HOME Study, a prospective pregnancy and birth cohort study from the Greater Cincinnati metropolitan area, enrolled women (N = 468) at 16 ± 3 weeks’ gestation between March 2003 and February 2006. Eligible women were those over 18 years, at no more than 19-weeks’ gestation, not taking medications for seizure or thyroid disorders, negative HIV status, without diagnosis of bipolar disorder, schizophrenia, diabetes or cancer that resulted in chemotherapy or radiation, and residing in housing built before 1978 as part of a study to assess lead hazards. Additional details about the broader study enrollment criteria, exposure measurements, and neurobehavioral assessments are published (Braun et al., 2017, 2020). We prospectively followed a total of 420 children (11 sets of twins included) completing at least one study visit from birth to age 12. At the age 12 study visit, we completed structural brain MRI examinations on 201 participants. While PFAS concentrations have been determined for HOME Study participants at multiple timepoints, for those who also completed MRI, we had the largest sample sizes for participants with PFAS concentrations during the prenatal period and age 12. We excluded 16 participants who had image artifacts from dental hardware, head motion and other technical factors (e.g., interference from a bias field, low signal to noise, etc.) that adversely impacted data quality. We also excluded one participant with a known congenital anomaly. With available imaging data from 184 participants, 22 were excluded due to missing gestational PFAS samples, and 26 were missing concurrent PFAS samples at the age 12 study visit. Maternal IQ was missing in 6 participants for the gestational analyses and in 4 participants at the age 12 analyses. Maternal pre-pregnancy BMI was missing for 1 participant for the gestational and age 12 analyses. This resulted in 155 adolescents for our analyses examining gestational PFAS and 153 examining age 12 PFAS concentrations. All data collection was completed prior to the onset of the COVID-19 pandemic.

### 2.2. Ethical considerations

The institutional review boards at Cincinnati Children’s Hospital Medical Center (CCHMC) and the enrolling delivery hospitals approved this study. The Centers for Disease Control and Prevention (CDC) laboratory’s involvement did not constitute engagement in human-subjects research. Pregnant women provided written informed consent for their own participation. Primary caregivers provided written informed consent for their child at the age 12 study visit, and adolescents provided written informed assent.

### 2.3. PFAS biomarker concentration

As previously reported (Fleury et al., 2024; Kuiper et al., 2024; Liu et al., 2024; Sultan et al., 2023), we collected venous blood samples from women during pregnancy at approximately 16-weeks and if unavailable, at 26-weeks of gestation (in the years 2003–2006), and from adolescents at the age 12 study visit (2016–2019). We separated serum from clotted venous blood samples and stored them at −80 °C prior to overnight shipment on dry ice to CDC. Laboratory staff quantified the serum concentrations of the following PFAS chemicals: perfluorooctanoic acid (PFOA), perfluorooctanesulfonic acid (PFOS), perfluorononanoic acid (PFNA), perfluorohexane sulfonic acid (PFHxS), and perfluorodecanoic acid (PFDA) using online solid phase extraction coupled to high-performance liquid chromatography-isotope dilution with tandem mass spectrometry (Kato et al., 2011; Kuklenyik et al., 2005). Each analytic batch included reagent blanks and low- and high-concentration QC samples. The limits of detection (LOD) for these PFAS chemicals were ~0.1 ng/mL (ranging from 0.082 to 0.2 ng/mL) and coefficients of variation between assays were ~6%; for values below the LOD, LOD/√2 was used instead (Hornung and Reed, 1990). Most PFAS were detectable in at least 98% of serum samples at both windows, except for PFDA, which was detected in 93.5% of pregnant women and 47.7% in their adolescents at age 12 study visit.

### 2.4. Imaging acquisition

Neuroimaging datasets for this study were acquired at CCHMC using a Philips Ingenia 3 T field strength MR System (Philips Healthcare, Best, The Netherlands) equipped with a 32-channel head coil. Anatomical MRI images of the brain were acquired using a three-dimensional, T1-weighted, fast Fourier echo sequence with a repetition time of 8 ms, echo time of 3.7 ms, flip angle of 8°, field of view dimensions of 256 mm (mm) by 224 mm by 192 mm, voxel size of 1 mm by 1 mm by 1 mm, 192 slices, sagittal slice orientation, a sensitivity encoding (SENSE) factor 2 (S reduction) applied and the sequence duration lasting 6 min, 20 s.

### 2.5. Image processing

T1-weighted images were processed using the Computational Anatomy Toolbox (CAT12, version 12.9 r2577, Structural Brain Mapping Group, Jena, Germany), which outputs scalar whole-brain measures to explore global effects (total gray matter volume, total white matter volume, total cerebral spinal fluid (CSF) volume, and average cortical thickness) as well as voxel- and surface-based maps to explore local effects on brain morphometry. Individual participants were mapped to the Montreal Neurological Institute’s (MNI) 152 standard brain template space (~1.5 mm spacing) using age-matched *a priori* tissue probability maps generated from the TOM8 toolbox for tissue segmentation. The resulting modulated, normalized gray matter volume maps were smoothed using a 6 mm full-width half-maximum Gaussian kernel and then used for voxel based morphometric (VBM) analyses. Next, the central surface morphometric measures (cortical thickness, sulcal depth) were determined using the projection-based thickness method. The central surface was then spatially registered to the FreeSurfer “FsAverage” template; measures of cortical thickness and sulcal depth were then projected onto the template space and smoothed along the surface with 12 mm and 20 mm full-width half-maximum Gaussian kernels, respectively, to be used for surface-based morphometry (SBM) analyses.

### 2.6. Statistical analyses

#### 2.6.1. Primary model

We conducted univariate analyses to examine data distribution and compute descriptive statistics for the PFAS concentrations and covariates. We compared characteristics of participants included in the current analysis with those excluded using chi-square test, *t*-test or Wilcoxon rank sum test, as appropriate. We log_2_-transformed PFAS concentrations to reduce the influence of extreme values. We set an arbitrary threshold of 60% detection of individual PFAS in participant samples for inclusion in the analytical models. We assessed correlations among log_2_-transformed serum PFAS concentrations using Pearson’s correlation. Imaging analyses were conducted using a general linear model framework implemented in CAT12 software version 12.9 (2577). A separate model was used to test for the association between the log_2_-transformed serum concentration of each PFAS during a specific time window (n = 9, five distinct PFAS measured at the second trimester of gestation and four distinct PFAS measured at age 12, as one PFAS did not exceed the detection threshold) and each morphometric measure. For each model, the morphometric measure was the dependent variable and the biological sex for the adolescent, adolescent race, household income at age 12, maternal full scale intelligence quotient (FSIQ), maternal pre-pregnancy BMI, primipara and total intracranial volume were included as covariates. For VBM and SBM analyses exploring spatially localized effects, variance explained by total intracranial volume at each voxel/vertex was regressed prior to model fitting. Statistical inferencing with false-discovery rate controlled at α = 0.05 was achieved for VBM and SBM analyses using the threshold-free cluster enhancement approach with 5000 permutations of the design matrix performed to estimate the null distribution (Gaser; Smith and Nichols, 2009). For models with significant associations, we examined post hoc the interaction with adolescent sex in the models using a significance threshold of p < 0.05. Whole brain volumes (gray, white and CSF) are measured in cubic centimeters (cm^3^), and cortical thickness in mm. VBM and SBM maps illustrate how much each voxel changes per doubling of PFAS concentration with units of mm^3^ and mm, respectively. Cohen’s f^2^ values were reported as a measure of effect size for the regression analyses to estimate the strength of explained variance to unexplained variance. Beta coefficients were reported in regression models to describe the expected change in the dependent variable for a one-unit change in an independent variable while holding other predictors constant. A 95% confidence interval (CI) for the regression beta coefficient provided a range of plausible values and indicated the precision of the estimated effect. The p-values for the coefficients were reported to indicate whether the relationships are statistically significant.

#### 2.6.2. Secondary model

As a secondary set of analyses, we examined the models using proximally focused covariates. For gestational age analyses, the covariates included maternal IQ, household income at baseline, maternal race, pre-pregnancy BMI, primipara, child sex and total intracranial volume as prescribed in the primary model. For age 12 analyses, the covariates included maternal IQ, household income at 12 y, adolescent race, adolescent BMI, adolescent sex and total intracranial volume as prescribed in the primary model.

#### 2.6.3. Sensitivity analysis of primary model

As a sensitivity analysis, we examined the model without maternal FSIQ included as a covariate.

## 3. Results

### 3.1. Participants

We analyzed data from participants with complete high-resolution, isotropic structural brain MRI, covariate data, and PFAS serum concentration for a given congener by exposure window (gestational (n = 155) or age 12 (n = 153)). Given that PFDA was detected in only 48% of the participant samples at age 12, we excluded it in analyses. We excluded participants with missing data (gestational (n = 265) or age 12 (n = 267)). The imaging study sample was predominately non-Hispanic White, on average aged 12 years (range 11–15.4 years), with median household incomes at age 12 of $75 K (interquartile range 35 K-125 K) and average maternal IQ (mean = 106). The characteristics of participants included in the current analyses were not significantly different from those not included (Table 1), except for age at study visit (p < 0.05), which was higher for participants not included in analyses for both exposure windows (p-value<0.001 for the comparison between the 155 participants in the gestational PFAS analysis and those 265 excluded; p-value = 0.002 for the comparison between the 153 participants in the age 12 PFAS analysis and those 267 excluded).

### 3.2. PFAS concentrations

**Fig. 1.**
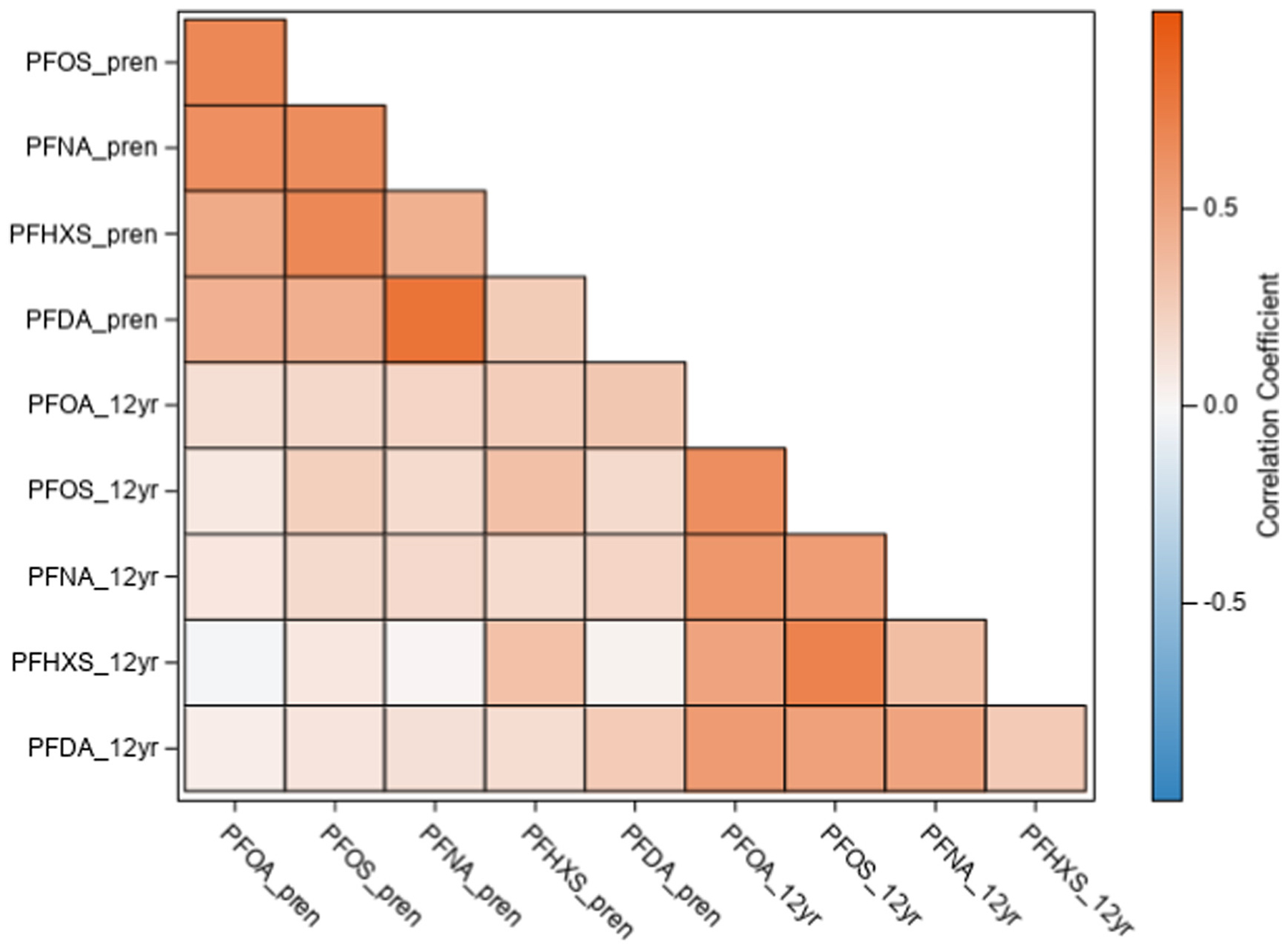
Pearson’s correlation coefficients of log_2_-transformed serum PFAS concentrations (perfluorooctanoic acid (PFOA), perfluorooctanesulfonic acid (PFOS), perfluorononanoic acid (PFNA), perfluorohexane sulfonic acid (PFHxS), and perfluorodecanoic acid (PFDA) for gestational (pren) and adolescent (12yr) windows.

PFOS had the highest median value of the 5 PFAS we measured, with a geometric mean of 12.8 ng/mL in the gestational window and 2.5 ng/mL at age 12 (Table 2). Consistently for all 5 PFAS, gestational serum concentrations were markedly higher than at age 12 (Table 2). Correlations among different PFAS within a single window were mostly moderate to strong (Pearson’s correlation coefficients = 0.26 to 0.82 for gestational window, 0.28 to 0.72 for age 12 window). However, for several PFAS congeners, correlations between gestational and age 12 windows were generally weak (Pearson’s correlation coefficients = 0.19, 0.25, 0.20, 0.34, 0.28 for PFOA, PFOS, PFNA, PFHxS and PFDA, respectively) (Fig. 1).

### 3.3. Whole-brain associations

A doubling in age 12 PFOA [*f*^*2*^ = 0.06, β = 10.79, (95% CI: 3.45, 18.13), p = 0.004], and PFNA [*f*^*2*^ = 0.04, β = 6.14, (95%CI: 1.09, 11.18), p = 0.018] concentrations were positively associated with total gray matter volume. Scatterplots illustrating the associations are shown in Supplemental Figs. 1 and 2. A doubling in age 12 PFOA [*f*^*2*^ = 0.04, β = −8.49, (95% CI: −15.28, −1.69), p = 0.015] and PFOS [*f*^*2*^ = 0.03, β = −5.00, (95% CI: −9.81, −0.18), p = 0.042] concentrations were negatively associated with total white matter volume. Scatterplots illustrating the associations are shown in Supplemental Figs. 3 and 4. A doubling in age 12 PFOA [(*f*^*2*^ = 0.03, β = 0.03, (95% CI: 0.00, 0.06), p = 0.034] and PFOS [(*f*^*2*^ = 0.03, β = 0.02, (95% CI: 0.00, 0.04), p = 0.035] concentrations was positively associated with whole brain average cortical thickness. Scatterplots illustrating the associations are shown in Supplemental Figs. 5 and 6. We found negligible effects on whole brain associations from the secondary and sensitivity analyses (Supplemental Tables 1 and 2). No significant associations were observed for gestational PFAS concentrations. Table 3 summarizes the Cohen’s f^2^ effect sizes and statistical significance of each PFAS concentration-whole-brain measure association. No sex-interactions were observed (Supplemental Table 3).

### 3.4. Voxel-based morphometry spatial associations

**Fig. 2.**
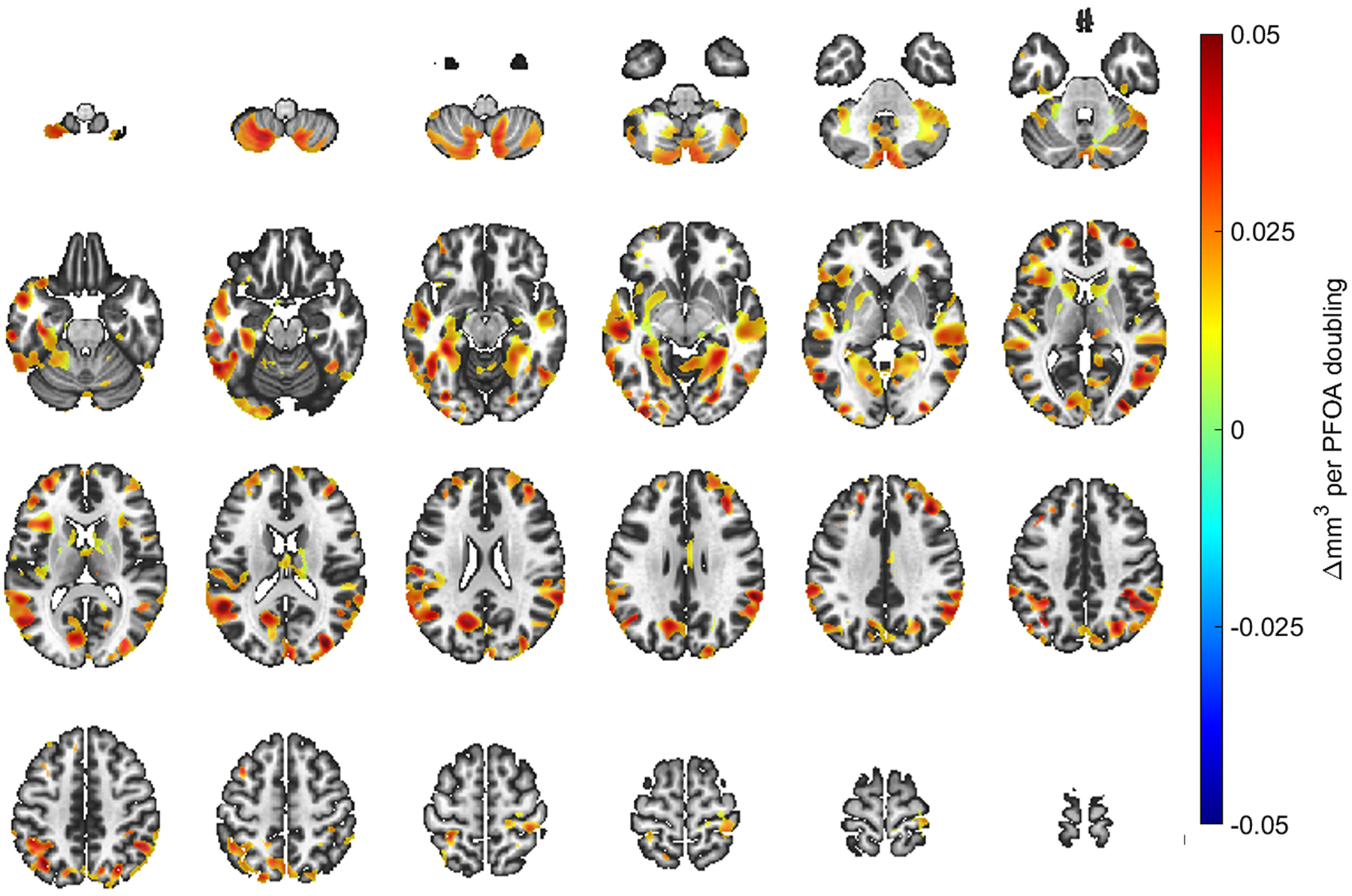
T1-weighted imaging template illustrating beta-coefficients of spatially localized regions demonstrating the associations identified from VBM analyses and perfluorooctanoic acid (PFOA) concentrations at the age 12 study visit. The template starts at the inferior aspect of the cerebellum with superior slices through the cerebrum to the cortical apex. As noted by the color scale, the change in mm^3^ corresponds to a doubling of the concurrent PFOA concentrations. Regions shown in the darkest red correspond to a 0.05 mm^3^ increase with a doubling of the concurrent PFOA concentration. The left side of an individual image corresponds to the left hemisphere to follow neurological convention.

**Fig. 3.**
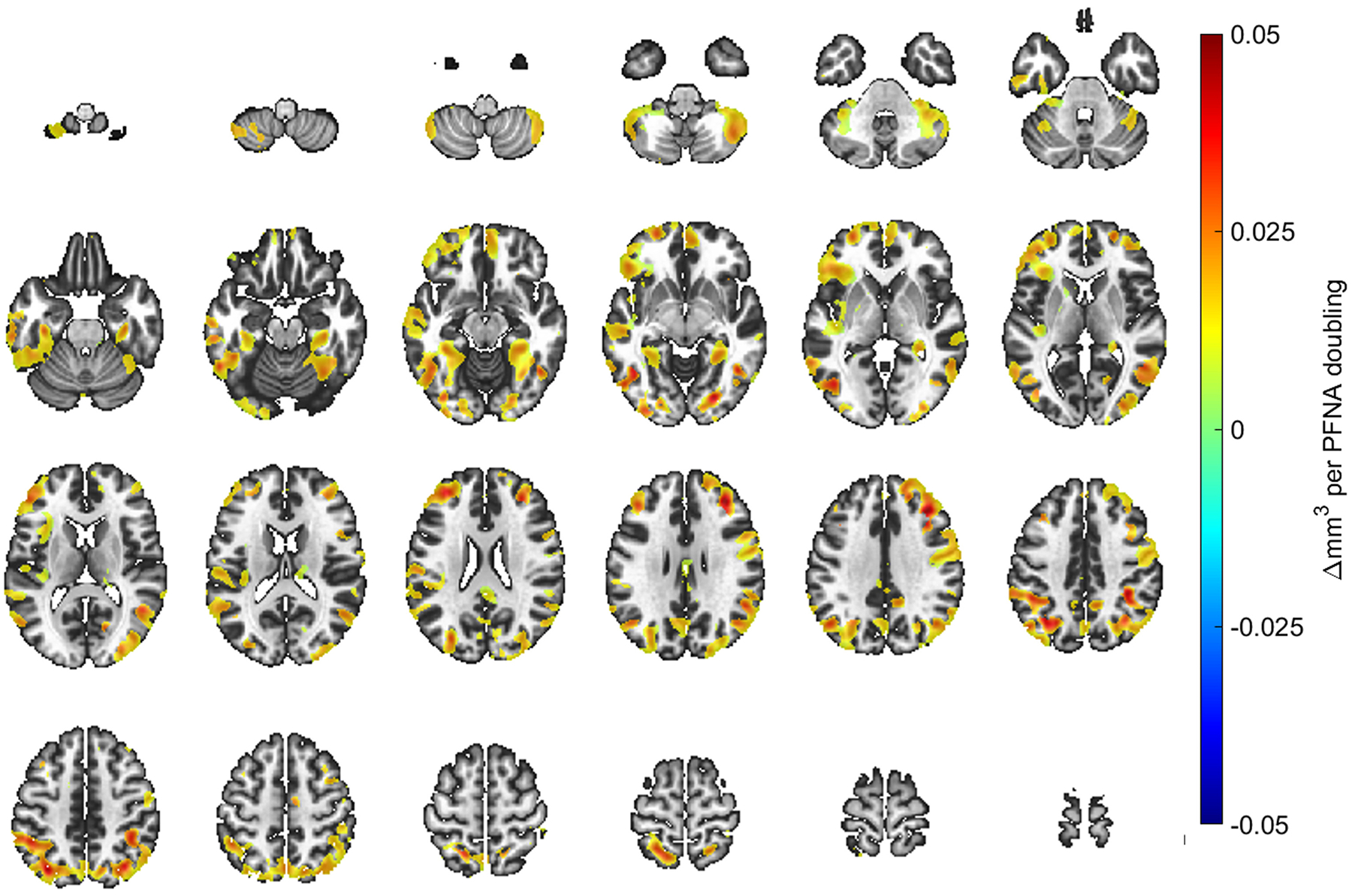
T1-weighted imaging template illustrating beta-coefficients of spatially localized regions demonstrating the associations identified from VBM analyses and perfluorononanoic acid (PFNA) concentrations at the age 12 study visit. The template starts at the inferior aspect of the cerebellum with superior slices through the cerebrum to the cortical apex. As noted by the color scale, the change in mm^3^ corresponds to a doubling of the concurrent PFNA concentrations. Regions shown in the darkest red correspond to a 0.05 mm^3^ increase with a doubling of the concurrent PFNA concentration. The left side of an individual image corresponds to the left hemisphere to follow neurological convention.

A doubling in age 12 PFOA and PFNA concentrations were positively associated with regionally localized, voxel-based gray matter volumes. Figs. 2 and 3 spatially illustrate regions where changes in gray matter volume with units of cubic millimeters were associated with a doubling in age 12 PFOA and PFNA concentrations, respectively. Associations were widespread with significant clusters appearing throughout every lobe of the neocortex and cerebellar volumes. While the associations were predominately cortical, subcortical and other gray matter regions such as the hippocampus, caudate nucleus and thalamus were also recognized, particularly for PFOA. The voxelwise t-stats between the primary and secondary models with proximally focused covariates have a correlation of 0.97. The sensitivity analyses removing maternal FSIQ yielded negligible effects on the results (Supplemental Figs. 8 and 9). No other PFAS congeners at either exposure window exhibited significant associations with gray matter volume when controlling for false-discovery rate α = 0.05 (Table 3). No sex-interactions were observed.

### 3.5. Surface based morphometry

**Fig. 4.**
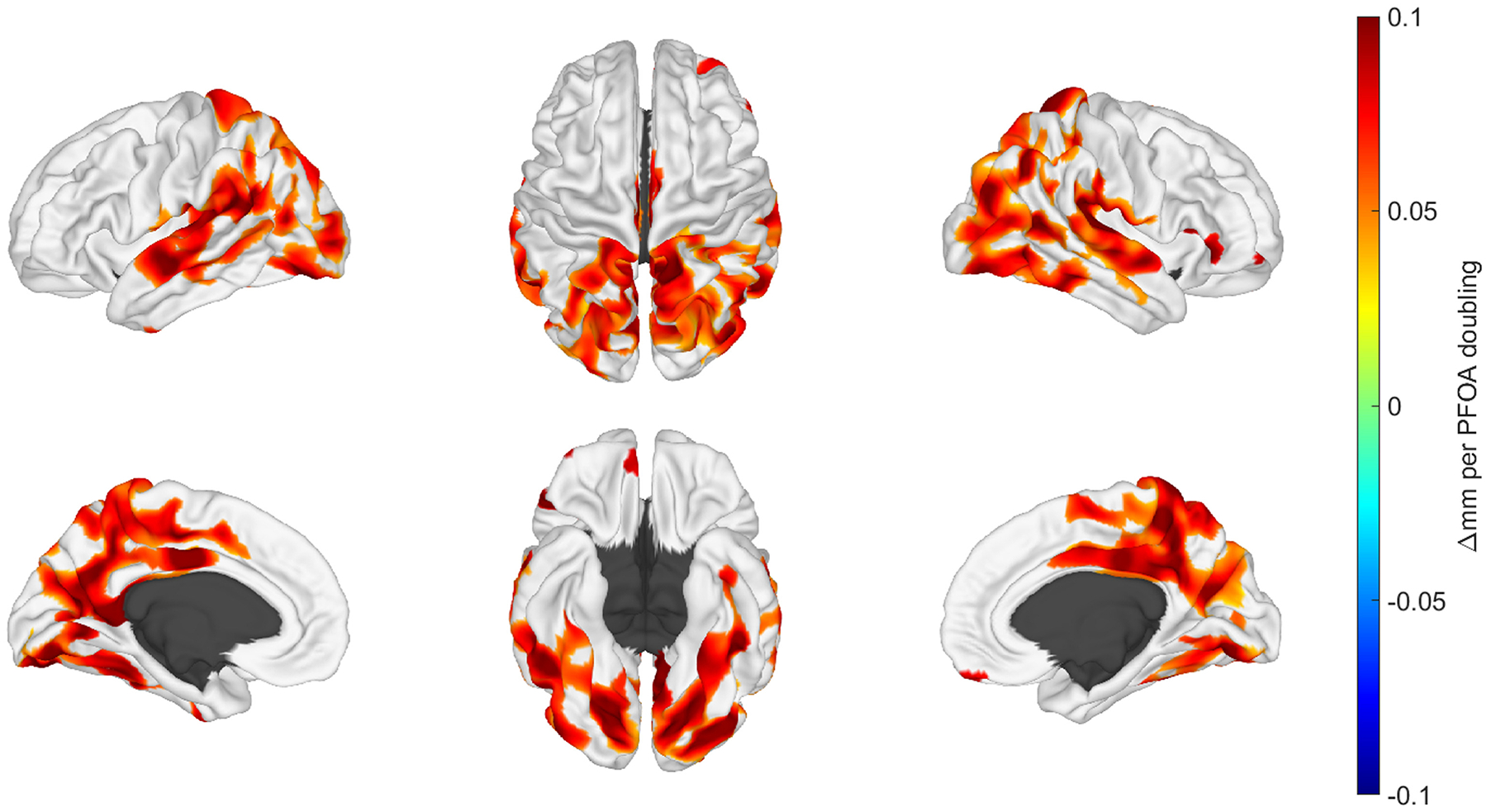
Illustrative brain renderings featuring beta-coefficients for regions where cortical thickness was associated with concurrent concentrations of perfluorooctanoic acid (PFOA) at the age 12 study visit. Top row from left to right represents the view of the left hemisphere, the superior view, and view of the right hemisphere. Bottom row from left to right represents the midline perspective of the left hemisphere, the inferior view and the midline view of the right hemisphere. As noted by the color scale, the change in mm corresponds to a doubling of the concurrent PFOA concentrations. Regions shown in the darkest red correspond to a 0.1 mm increase with a doubling of the concurrent PFOA concentration.

**Fig. 5.**
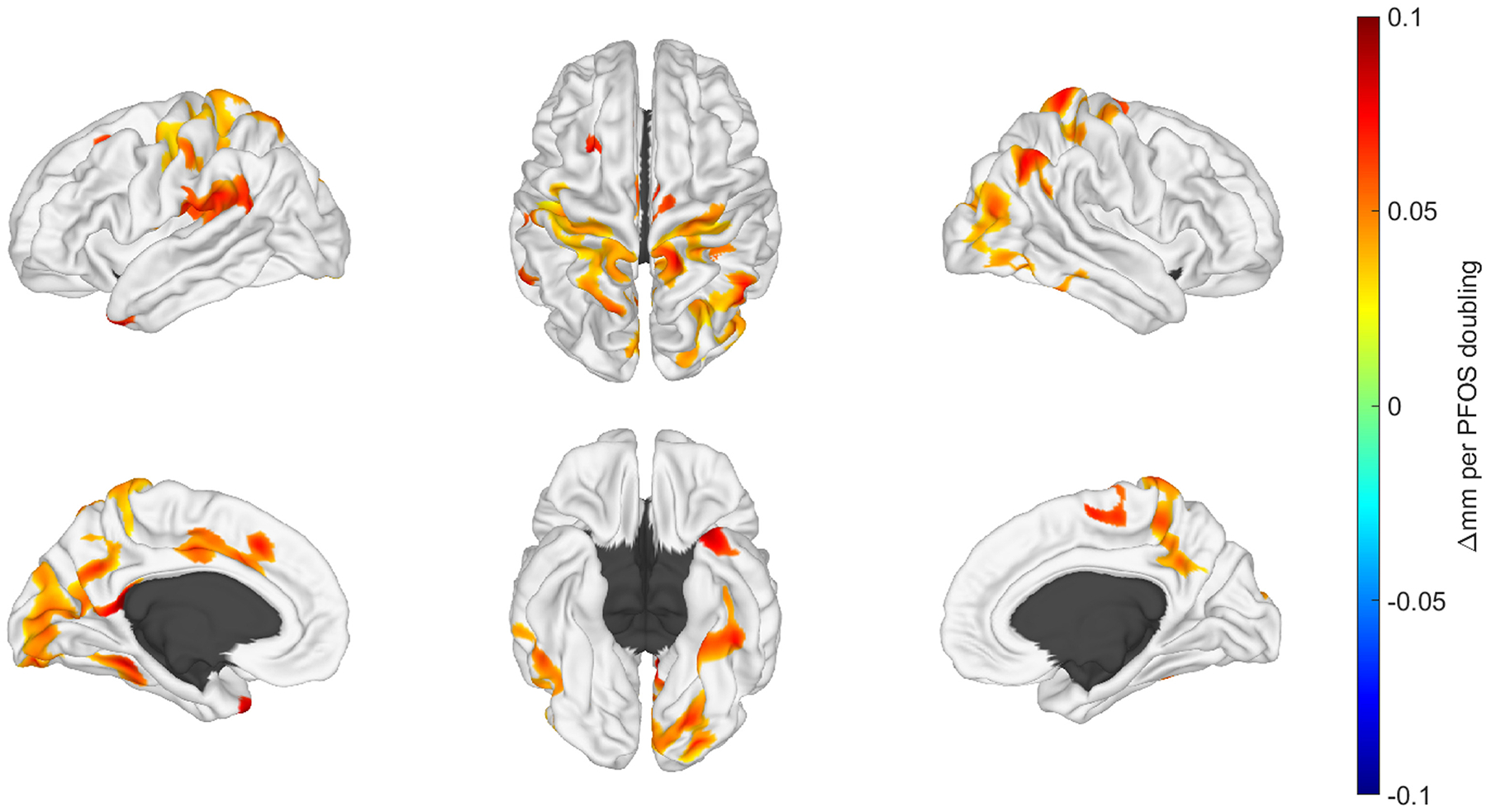
Illustrative brain renderings featuring beta-coefficients for regions where cortical thickness was associated with concurrent perfluorooctanesulfonic acid (PFOS) concentrations at the age 12 study visit. Top row from left to right represents the view of the left hemisphere, the superior view, and view of the right hemisphere. Bottom row from left to right represents the midline perspective of the left hemisphere, the inferior view and the midline view of the right hemisphere. As noted by the color scale, the change in mm corresponds to a doubling of the concurrent PFOS concentrations. Regions shown in the darkest red correspond to a 0.1 mm increase with a doubling of the concurrent PFOS concentration.

Regionally localized, surface-based measures of cortical thickness were positively associated with a doubling in age 12 PFOA and PFOS concentrations. For both congeners, significant clusters were predominantly located in the posterior aspects of the brain in the temporal, parietal, and occipital lobes. Figs. 4 and 5 spatially illustrate regions where changes in cortical thickness were associated with doubling in age 12 PFOA and PFOS concentrations, respectively.

We found negligible effects on cortical thickness associations from the secondary and sensitivity analyses (Supplemental Figures 10–13). No other PFAS congeners at either exposure window exhibited significant associations with cortical thickness when controlling false-discovery rate α = 0.05. We also examined un-threshold maps to explore any convergence with the study by Barron et al. (2025). We identified no significant associations between sulcal depth and any PFAS congeners at either exposure window. No sex-interactions were observed.

## 4. Discussion

Our analyses show that higher PFAS concentrations measured in early adolescence were associated with larger concurrent structural volumes and cortical thickness. While this finding may be unexpected for a class of chemicals with demonstrated toxicity in a variety of domains (endocrine, immune, liver, kidney and brain development) (Starnes et al., 2022), our findings during this period of development may represent delays in typical brain maturation, disruption of neuronal activities, PFAS accumulation itself, or possibly some combination of these purported mechanisms of action.

The positive associations with gray matter may signify that a higher proportion of the intracranial space was occupied by gray matter. The corresponding inverse relationship of concurrent PFOA and PFOS serum concentrations with less white matter volume may also provide support for delayed maturation. Typically, adolescents in this age range demonstrate lesser amounts of cortical gray matter and more white matter (Giedd et al., 1999; Lebel and Beaulieu, 2011; Ostby et al., 2009; Shaw et al., 2008; Tamnes et al., 2010) as this reflects developmental pruning. Greater gray matter volume and cortical thickness may reflect delayed synaptic pruning (Nakua et al., 2025). Liao et al. (2008) demonstrated PFOS-induced changes in calcineurin activity impeded synaptic pruning with in vitro models of hippocampal neurons. In a model of rat neural stem cell (NSC)-derived neurons, exposure to PFOS and PFOA produced morphological alterations with a decrease in cell body area, however, with PFOS increasing the NSC proliferation (Pierozan and Karlsson, 2021). Exposure to 1 and 10 μM PFOA also affected the neurite network and caused an increase in the number of processes and branches per cell (Pierozan and Karlsson, 2021). These findings suggest multiple mechanisms are involved with PFAS effects on the brain.

Associations of PFAS concentrations during the gestational period and morphometric features at adolescence were largely absent in the HOME Study cohort. In contrast, Barron et al. reported that select gestational PFAS plasma concentrations predicted a component involving the occipital cortex volume and surface area when simultaneously evaluating multi-modal MRI features with independent component analyses (Barron et al., 2025) using a subset of the FinnBrain Birth Cohort. Branched PFHxS had a negative association, however, branched PFOA showed a positive association. Our findings illustrated in Fig. 4 demonstrate high spatial convergence with cortical morphology in the posterior portions shown for PFOA in Barron et al. (2025). We explored if our thresholding limited our ability to find similar spatial associations (Supplemental Figs. 14 and 15). For PFOA, the effect size (mm/doubling) in non-significant regions of the occipital cortex is not very strong, however, there is a modest effect in non-significant regions of the inferior frontal lobe that does bear similarity to pattern observed by Barron et al. (2025). We point out the distinction that the Barron et al. findings are for cortical surface area and not cortical thickness. For PFOS, we observed some modest but non-significant effects in the occipital and in the inferior frontal lobes. However, there were key differences between their study design including the PFAS composition and concentrations with ranges of 0–1 ng/mL for the European based cohort and the mean age of the children at 5.4 years representing a different stage of development and brain maturation. In another environmental imaging study, Wang et al. evaluated associations of PFAS and phthalic acid esters with multi-modal imaging in 52 adolescents from a birth cohort longitudinally followed in Taiwan. At age 14–15 years, adolescents completed an MRI examination with high-resolution anatomical and multi-shell diffusion imaging. Morphometric analyses employed FreeSurfer (Fischl, 2012) postprocessing with Bonferroni correction for multiple comparisons and focused exclusively on cortical findings by reporting surface area and thickness with no analyses reported for volumes of white matter, deep gray or other structures. Prenatal PFDA from cord blood demonstrated a positive relationship with cortical surface area with the left postcentral gyrus, adjusting for sex and family income. When stratifying by sex, males demonstrated a positive relationship of cord blood PFDA with cortical surface area with the left rostral anterior cingulate cortex, adjusting for family income. Males also showed an inverse correlation of cord blood PFOA with cortical thickness in the pars triangularis region of the right inferior frontal gyrus. For female adolescents, no significant PFAS associations were observed. The Wang et al. study features adolescent imaging, however, there is little concordance between our study and with their spatial associations for morphometry, likely due to differences in the PFAS exposure assessment and post-processing methodology. However, our strongest associations for the gestational window, though not reaching significance, also featured maternal serum PFDA. While our study focused completely on morphometric findings, the study from Taiwan also examined diffusion imaging metrics which revealed inverse associations for PFDA fiber track integrity metrics for cerebellar peduncles, anterior thalamic radiation, anterior commissure, and corpus callosum in adolescents adjusted for family income. These findings showed little agreement with another environmental imaging study employing diffusion imaging also arising from different exposure assessments and post-processing methodologies. England-Mason et al. reported higher second trimester maternal PFAS plasma concentrations were associated with altered white matter microstructure for a sub-cohort of the Alberta Pregnancy Outcomes and Nutrition Study evaluated from 2 to 6 years of age with longitudinal diffusion tensor imaging (England-Mason et al., 2025). In the body and splenium of the corpus callosum, maternal perfluoroalkyl sulfonates were associated with tract-based diffusion metrics indicating lower fractional anisotropy and higher mean diffusivity values with these findings also interpreted as reflecting maturation delays. In females, higher maternal PFOS concentrations were associated with less mature white matter diffusion metric patterns of the fornix, but more mature patterns for pyramidal fibers. Less mature patterns of the superior longitudinal fasciculus were noted in males associated with maternal PFHxS concentrations. While the current study is consistent with the findings of England-Mason et al. with maturation delays, the imaging methodologies, participant ages, exposure windows and other characteristics are different.

While maturation delays provide a plausible explanation, the findings may also support other mechanisms such as accumulation of PFAS in the brain. Besides the widespread cortical associations, we also appreciated regionally localized, voxel-based volumetric associations in deep gray matter including the thalamus, hippocampus and caudate nucleus, especially for PFOA along with some possible globus pallidus involvement. Di Nisio et al. (Di Nisio et al., 2022) reported PFOA, PFHxS and PFHxA concentrations upon post-mortem examination of select brain specimens from five middle aged males living in an Italian region with known PFAS environmental pollution. Their analyses found that regions of the basal ganglia, specifically the hypothalamus, caudate nucleus and lenticular nucleus (which includes globus pallidus and putamen) demonstrated the highest concentrations of PFAS for their sample. They suggested a gradient-driven trans-mural diffusion across the blood brain barrier for PFAS accumulation (Di Nisio et al., 2022). Suzuki et al. (2025) reported the presence of four of the five PFAS we measured in their post-mortem brain specimens obtained from older adults (56–79 years) though PFOA was not detected. They mentioned that differences in the brain/serum ratios of various PFAS are not due to residual blood but arise from PFAS partitioning across the blood-brain barrier into brain tissue. Their analysis found that PFAS with more carbon atoms have a greater propensity to partition into brain tissue. No differences in regional brain tissue PFAS concentrations were observed when comparing only two regions, the temporal pole and middle frontal cortices. Suzuki et al. stated their investigation of different PFAS accumulation patterns in cortical regions was based upon the sampling of brain specimens from polar bears by Greaves et al. (2013). Eggers Pedersen et al. observed in agreement with Greaves et al. (2013) and postulated that hindbrain regions of polar bears in Greenland received the incoming bloodstream and if contaminated with PFAS, then these regions would first accumulate PFAS in higher amounts relative to other regions (Eggers Pedersen et al., 2015). Positive correlations between PFAS and monamine oxidase activity and glutamine synthetase activity were observed in the occipital lobe with inverse correlations between PFAS concentrations in the frontal cortex, thalamus and cerebellum and acetylcholinesterase activity (Eggers Pedersen et al., 2015).

Studies featuring brain morphometry often examine structural alterations to explain psychiatric and/or neurobehavioral outcomes, such as externalizing and internalizing symptoms, autism spectrum disorders and attention deficit hyperactivity disorders (ADHD) (Nakua et al., 2025; Sharp et al., 2023; Wu et al., 2023). For example, the literature on pediatric ADHD has been increasing populated with analyses using data from the Adolescent Brain and Cognitive Development (ABCD) Study with FreeSurfer outputs for volume, cortical surface area and thickness (Batty et al., 2010; Bernanke et al., 2022; Dall’Aglio et al., 2022; Owens et al., 2021; Reimann et al., 2024; Rosch et al., 2024; Sarabin et al., 2023). Meta-analyses for ADHD do not report convergence of findings (Hoogman et al., 2019; Samea et al., 2019). The alterations described in our current study are not congruent with the features often reported in ADHD that include lower gray matter volumes, stable white matter volumes with reduced cortical thickness or surface area. However, longitudinal multimodal imaging analyses are required to address the effects of PFAS and determine neurobehavioral associations in humans.

### 4.1. Strengths and limitations

Our study has several strengths. Foremost is the prospective pregnancy and birth cohort design with longitudinal follow-up as it allowed us to examine gestational and concurrent PFAS concentrations with high-resolution anatomical imaging data during early adolescence. We employed distinct neuromorphometric approaches in our study to allow for non-biased assessment of morphometric features. VBM is a highly popular method that relies on the creation of probabilistic maps of gray matter, white matter, and cerebrospinal fluid using models based on the voxel signal intensity and known spatial definitions. However, VBM cannot differentiate between variations in gray matter density caused by differences in cortical thickness versus differences in gyrification or folding. Meanwhile, SBM is a method that addresses some of the limitations of VBM. In this technique, individual cortical surfaces are mapped onto a spherical template surface. SBM extracts the outer brain surface and gray/white interface, enabling precise estimation of cortical thickness while avoiding confusion between thickness and gyrification of gray matter. While we accounted for multiple testing across voxel in each model, we did not adjust for multiple testing between models as each outcome is derived from the same data, so this must be acknowledged as a limitation.

The HOME Study represents a metropolitan community within the Midwest region of the United States with a typically developing cohort consisting of urban, suburban and rural residents. The HOME Study enrolled pregnant women at 16 weeks’ gestation between 2003 and 2006. For those unable to provide a sample at enrollment, we also had sample collection at 26 weeks. For the current study, we decided to maximize the number of participants, but had only 12 participants for whom we used the 26 weeks’ PFAS serum concentrations. Given the estimated PFAS half-lives that we investigated are on the order of years, we regarded the sampling in the second trimester as a good representation for gestational exposure of the mother. However, this second semester sampling limited the ability to investigate the impact of the PFAS exposure across other trimesters. The median values of most PFAS concentrations for the HOME Study participants were comparable with other study populations in the United States (Calafat et al., 2007; Jackson-Browne et al., 2020; Pinney et al., 2014). However, the HOME Study participants resided in the greater Cincinnati, Ohio region with historical PFOA contamination, which may have influenced their exposure and vulnerability (Pinney et al., 2014). Also, enrollment criteria for the original design of the HOME Study required mothers to live in housing constructed prior to 1978, so the findings from these analyses may be less generalizable to other populations. Most of the HOME study participants were non-Hispanic White, with relatively high household incomes so the results may not be generalizable to more heterogeneous populations. Like other birth cohorts, the HOME Study experienced attrition at the age 12 study visit. There is a potential for selection bias, as we excluded 267 adolescents with missing data or did not complete the MRI examination. While the number of participants in this study is relatively large for a single site morphometric MRI analysis of a pediatric environmental cohort and may be the largest PFAS-focused neuroimaging study published to date, it remains a modest sample size for an epidemiology study. The work currently features only one imaging examination timepoint that occurred in early adolescence and concurrent with the age 12 PFAS exposure assessment, which may be subject to limitations of a cross-sectional analysis. Changes in the associations arising from covariate selections in secondary and sensitivity analyses were negligible in comparison to our primary model. We retained maternal FSIQ in our primary model for comparability with other analyses in the HOME Study. Maternal FSIQ has been used as a covariate in analyses of other neurotoxicants as it captures a variety of factors. Our study is also limited as we did not examine PFAS mixtures or perform adjustments in the models for other PFAS co-exposures. Residual confounding from other environmental exposures not considered in the models is another possible limitation. Future studies that evaluate PFAS and other environmental exposures may yield a more comprehensive understanding of health risks associated with PFAS exposures.

## 5. Conclusions

In early adolescence, we found that concurrent serum PFAS concentrations were associated with increases in brain volume and cortical thickness. No significant associations were observed for gestational PFAS concentrations. The significance of these associations is uncertain, but the findings deviate from those expected with typical development and may reflect delays in brain maturation. Such findings may explain neurobehavioral deficits in cognition, language and motor development in infancy, and increased attention problems, hyperactivity and reduced ability to perform activities of daily living in childhood as previously reported in the literature (Ames et al., 2025; Vuong et al., 2021b). Continued research is essential to assess whether these associations persist or evolve into adulthood. Further investigations exploring the significance of the findings are needed along with contributions from other environmental exposures.

## Supplementary Material

Supplementary Material-Tables

Supplementary Material-Figures

## Figures and Tables

**Table 1.** Characteristics of Health Outcomes and Measures of the Environment (HOME) Study Participants

	All 420	In gestational analysis (n=155)	Not in gestational analysis (n=265)	In 12-yr analysis (n=153)	Not in 12-yr analysis (n=267)
Adolescent sex, female	226 (53.8%)	89 (57.4%)	137 (51.7%)	83 (54.3%)	143 (53.6%)
Adolescent race					
Black	140 (33.7%)	55 (35.5%)	85 (32.7%)	59 (38.6%)	81 (30.9%)
Non-Black	275 (66.3%)	100 (64.5%)	175 (67.3%)	94 (61.4%)	181 (69.1%)
Age at MRI/study visit, y	12.5 (0.9)	12.3 (0.8)	13.2 (1)	12.4 (0.8)	12.9 (1.1)
Household income at age 12 visit ($ thousand)	75 (35, 130)	75 (35, 130)	75 (35, 155)	75 (35, 125)	90 (35, 155)
Maternal FSIQ	106.2 (14.7)	106.3 (15.6)	106.2 (14.1)	105.8 (15.9)	106.4 (13.9)
Pre-pregnancy BMI	26.2 (6.3)	26.7 (6.6)	25.9 (6.1)	26.5 (6.4)	26.1 (6.2)
Primipara	178 (44.0%)	59 (38.1%)	119 (47.6%)	55 (35.9%)	123 (48.8%)

Arithmetic mean values and standard deviations are reported for age at MRI, maternal full scale intelligence quotient (FSIQ), maternal pre-pregnancy body mass index (BMI) and primipara. Medians (25^th^ percentile, 75^th^ percentile) are reported for household income. Numbers, and percentages of sub-cohorts are reported for other characteristics. Missingness for all 420 included adolescent race (n=5), age at MRI/study visit (n=164), household income at age 12 visit (n=164), maternal FSIQ (n= 16), pre-pregnancy BMI (n=22), and primipara (n=15).

**Table 2.** Serum PFAS Concentrations (ng/mL)

Exposure window	PFAS	N	Minimum	Maximum	P25	Median	P75	Geomean (95%CI)	%LOD
Gestational	PFOA	155	0.5	17.4	3.6	5.2	7.5	5.2 (4.8, 5.6)	100
	PFOS	155	0.4	48.7	9.1	13.3	18.7	12.8 (11.7, 14.0)	100
	PFNA	155	0.1	2.9	0.7	0.9	1.2	0.9 (0.8, 1)	100
	PFHxS	155	<LOD	10.7	0.8	1.3	2.3	1.4 (1.2, 1.6)	99.4
	PFDA	155	<LOD	1.3	0.1	0.2	0.3	0.2 (0.2, 0.2)	93.6
Age 12	PFOA	153	0.47	5.17	1.0	1.3	1.6	1.3 (1.2, 1.3)	100
	PFOS	153	0.9	11.9	1.8	2.4	3.4	2.5 (2.3, 2.7)	100
	PFNA	153	<LOD	2.5	0.2	0.3	0.5	0.3 (0.3, 0.4)	98.7
	PFHxS	153	<LOD	20.3	0.5	0.7	1.0	0.7 (0.7, 0.8)	99.4
	PFDA	153	<LOD	0.5	<LOD	<LOD	0.1	0.1 (0.1, 0.1)	48.1

SD: standard deviation; P25: 25^th^ percentile; P75: 75^th^ percentile; Geomean: geometric mean; 95%CI: 95% confidence Interval; LOD: limit of detection; perfluorooctanoic acid (PFOA), perfluorooctanesulfonic acid (PFOS), perfluorononanoic acid (PFNA), perfluorohexane sulfonic acid (PFHxS), and perfluorodecanoic acid (PFDA)

**Table 3.** Associations of log_2_-transformed serum PFAS concentrations with whole-brain morphometric measurements, adjusting for adolescent sex, adolescent race, household income at age 12 study visit, maternal IQ, maternal pre-pregnancy BMI, primipara and total intracranial volume for each exposure window. Cohen’s effect sizes (f^2^), p-values, Beta coefficients (β) and 95% confidence intervals are presented. Volumes of gray matter, white matter and cerebral spinal fluid (CSF) units are in cubic centimeters. Cortical thickness units are in millimeters. PFAS abbreviations: perfluorooctanoic acid (PFOA), perfluorooctanesulfonic acid (PFOS), perfluorononanoic acid (PFNA), perfluorohexane sulfonic acid (PFHxS), and perfluorodecanoic acid (PFDA).

			Gray Matter			White Matter			CSF			Average Cortical Thickness		
Exposure Window	PFAS	N	*f* ^*2*^	β (95% C.I.)	p-value	*f* ^*2*^	β (95% C.I.)	p-value	*f* ^*2*^	β (95% C.I.)	p-value	*f* ^*2*^	β (95% C.I.)	p-value
Gestational	PFOA	155	0	1.335 (−3.6246, 6.2945)	0.596	0	−1.909 (−6.4463, 2.6293)	0.407	0	0.574 (−4.4002, 5.5473)	0.820	0	0.005 (−0.0148, 0.0250)	0.615
	PFOS	155	0	0.738 (−3.7125, 5.1886)	0.744	0	0.617 (−3.4612, 4.6948)	0.765	0	−1.355 (−5.8107, 3.1010)	0.549	0	−0.002 (−0.0198, 0.0160)	0.834
	PFNA	155	0.01	4.213 (−1.5093, 9.9350)	0.148	0.02	−3.945 (−9.1860, 1.2963)	0.139	0	−0.268 (−6.0432, 5.5072)	0.927	0.01	0.016 (−0.0074, 0.0386)	0.182
	PFHXS	155	0	−0.991 (−4.4675, 2.4856)	0.574	0.02	2.752 (−0.4046, 5.9091)	0.087	0.01	−1.761 (−5.2368, 1.7143)	0.318	0	−0.003 (−0.0169, 0.0110)	0.678
	PFDA	155	0.02	4.301 (−0.2405, 8.8430)	0.063	0.02	−3.657 (−7.8253, 0.5111)	0.085	0	−0.644 (−5.2483, 3.9600)	0.783	0.02	0.017 (−0.0008, 0.0357)	0.060
Age 12	PFOA	153	***0.06***	***10.791 (3.4538, 18.1291)***	***0.004***	***0.04***	−***8.487 (−15.2812, −1.6932)***	***0.015***	0	−2.304 (−9.1954, 4.5868)	0.510	***0.03***	***0.033 (0.0025, 0.0629)***	***0.034***
	PFOS	153	0.02	4.918 (−0.3373, 10.1743)	0.066	***0.03***	−***4.999 (−9.8146, −0.1830)***	***0.042***	0	0.080 (−4.7808, 4.9415)	0.974	***0.03***	***0.023 (0.0017, 0.0442)***	***0.035***
	PFNA	153	***0.04***	***6.135 (1.0880, 11.1816)***	***0.018***	0.01	−2.469 (−7.1799, 2.2424)	0.302	0.02	−3.666 (−8.3321, 1.0000)	0.123	0.01	0.015 (−0.0062, 0.0354)	0.167
	PFHXS	153	0	0.426 (−3.0584, 3.9108)	0.809	0	−0.451 (−3.6524, 2.7500)	0.781	0	0.025 (−3.1610, 3.2110)	0.988	0	0.002 (−0.0123, 0.0160)	0.797

## Data Availability

Data will be made available on request.

## Appendix A. Supplementary data

Supplementary data to this article can be found online at https://doi.org/10.1016/j.envres.2026.124338.

## References

[R1] Ames JL, , 2025. Effects of early-life PFAS exposure on child neurodevelopment: a review of the evidence and research gaps. Curr. Environ. Health Rep 12, 9. 39888511 10.1007/s40572-024-00464-5 PMC11785707

[R2] Appel M, , 2022. The transplacental transfer efficiency of per- and polyfluoroalkyl substances (PFAS): a first meta-analysis. J. Toxicol. Environ. Health B Crit. Rev 25, 23–42. 34930098 10.1080/10937404.2021.2009946

[R3] ATSDR, 2021. Toxicological profile for perfluoroalkyls. In: U.S. Department of Health and Human Services Agency for Toxic Substances and Disease Registry (ATSDR) EPA-HQ-OLEM-2023-0278-0019. 37220203

[R4] Barron A, , 2025. Prenatal exposure to perfluoroalkyl substances predicts multimodal brain structural and functional outcomes in children aged 5 years: a birth cohort study. Lancet Planet. Health, 101309. 41077058 10.1016/j.lanplh.2025.101309

[R5] Batty MJ, , 2010. Cortical gray matter in attention-deficit/hyperactivity disorder: a structural magnetic resonance imaging study. J. Am. Acad. Child Adolesc. Psychiatry 49, 229–238. 20410712 10.1016/j.jaac.2009.11.008 PMC2829134

[R6] Bernanke J, , 2022. Structural brain measures among children with and without ADHD in the adolescent brain and cognitive development study cohort: a cross-sectional US population-based study. Lancet Psychiatry 9, 222–231. 35143759 10.1016/S2215-0366(21)00505-8

[R7] Bharal B, , 2024. Neurotoxicity of per- and polyfluoroalkyl substances: evidence and future directions. Sci. Total Environ 955, 176941. 39454776 10.1016/j.scitotenv.2024.176941

[R8] Braun JM, , 2020. Adolescent follow-up in the health outcomes and measures of the environment (HOME) study: cohort profile. BMJ Open 10, e034838. 10.1136/bmjopen-2019-034838 PMC7228515 32385062

[R9] Braun JM, , 2017. Cohort profile: the health outcomes and measures of the environment (HOME) study. Int. J. Epidemiol 46, 24. 27006352 10.1093/ije/dyw006 PMC5837495

[R10] Buck RC, , 2011. Perfluoroalkyl and polyfluoroalkyl substances in the environment: terminology, classification, and origins. Integrated Environ. Assess. Manag 7, 513–541. 10.1002/ieam.258 PMC3214619 21793199

[R11] Calafat AM, , 2007. Polyfluoroalkyl chemicals in the U.S. population: data from the national health and nutrition examination survey (NHANES) 2003–2004 and comparisons with NHANES 1999–2000. Environ. Health Perspect 115, 1596–1602. 18007991 10.1289/ehp.10598 PMC2072821

[R12] Cao Y, Ng C, 2021. Absorption, distribution, and toxicity of per- and polyfluoroalkyl substances (PFAS) in the brain: a review. Environ. Sci. Process. Impacts 23, 1623–1640. 34533150 10.1039/d1em00228g

[R13] Dall’Aglio L, , 2022. Attention-deficit hyperactivity disorder symptoms and brain morphology: examining confounding bias. eLife 11. 10.7554/eLife.78002 PMC9708072 36350121

[R14] De Silva AO, , 2021. PFAS exposure pathways for humans and wildlife: a synthesis of current knowledge and key gaps in understanding. Environ. Toxicol. Chem 40, 631–657. 33201517 10.1002/etc.4935 PMC7906948

[R15] Di Nisio A, , 2022. Impairment of human dopaminergic neurons at different developmental stages by perfluoro-octanoic acid (PFOA) and differential human brain areas accumulation of perfluoroalkyl chemicals. Environ. Int 158, 106982. 34781208 10.1016/j.envint.2021.106982

[R16] Domingo JL, 2025. A review of the occurrence and distribution of Per- and polyfluoroalkyl substances (PFAS) in human organs and fetal tissues. Environ. Res 272, 121181. 39978621 10.1016/j.envres.2025.121181

[R17] Eggers Pedersen K, , 2015. Brain region-specific perfluoroalkylated sulfonate (PFSA) and carboxylic acid (PFCA) accumulation and neurochemical biomarker responses in east Greenland polar bears (Ursus maritimus). Environ. Res 138, 22–31. 25682255 10.1016/j.envres.2015.01.015

[R18] England-Mason G, , 2025. Maternal concentrations of perfluoroalkyl sulfonates and alterations in white matter microstructure in the developing brains of young children. Environ. Res 267, 120638. 39681179 10.1016/j.envres.2024.120638

[R19] Fischl B, 2012. FreeSurfer. Neuroimage 62, 774–781. 22248573 10.1016/j.neuroimage.2012.01.021 PMC3685476

[R20] Fleury ES, , 2024. Evaluating the association between longitudinal exposure to a PFAS mixture and adolescent cardiometabolic risk in the HOME study. Environ. Epidemiol 8, e289. 38343730 10.1097/EE9.0000000000000289 PMC10852393

[R21] Gaser C. Threshold free cluster enhancement toolbox. http://dbm.neuro.uni-jena.de/tfce/.

[R22] Giedd JN, , 1999. Brain development during childhood and adolescence: a longitudinal MRI study. Nat. Neurosci 2, 861–863. 10491603 10.1038/13158

[R23] Glüge J, , 2020. An overview of the uses of per- and polyfluoroalkyl substances (PFAS). Environ. Sci. Process. Impacts 22, 2345–2373. 33125022 10.1039/d0em00291g PMC7784712

[R24] Greaves AK, , 2013. Brain region distribution and patterns of bioaccumulative perfluoroalkyl carboxylates and sulfonates in east Greenland polar bears (Ursus maritimus). Environ. Toxicol. Chem 32, 713–722. 23280712 10.1002/etc.2107

[R25] Gyllenhammar I, , 2018. Perfluoroalkyl acids (PFAAs) in serum from 2–4-Month-Old infants: influence of maternal serum concentration, gestational age, breast-feeding, and contaminated drinking water. Environ. Sci. Technol 52, 7101–7110. 29758986 10.1021/acs.est.8b00770

[R26] Hoogman M, , 2019. Brain imaging of the cortex in ADHD: a coordinated analysis of large-scale clinical and population-based samples. Am. J. Psychiatr 176, 531–542. 31014101 10.1176/appi.ajp.2019.18091033 PMC6879185

[R27] Hornung R, Reed LD, 1990. Estimation of average concentration in the presence of nondetectable values. Appl. Occup. Environ. Hyg 5, 46–51.

[R28] Jackson-Browne MS, , 2020. PFAS (per- and polyfluoroalkyl substances) and asthma in young children: NHANES 2013–2014. Int. J. Hyg Environ. Health 229, 113565. 32485600 10.1016/j.ijheh.2020.113565 PMC7492379

[R29] Jia Y, , 2022. Insights into the competitive mechanisms of Per- and polyfluoroalkyl substances partition in liver and blood. Environ. Sci. Technol 56, 6192–6200. 35436088 10.1021/acs.est.1c08493

[R30] Kato K, , 2011. Improved selectivity for the analysis of maternal serum and cord serum for polyfluoroalkyl chemicals. J. Chromatogr. A 1218, 2133–2137. 21084089 10.1016/j.chroma.2010.10.051

[R31] Kuiper JR, , 2024. Estimating effects of longitudinal and cumulative exposure to PFAS mixtures on early adolescent body composition. Am. J. Epidemiol 193, 917–925. 38400650 10.1093/aje/kwae014 PMC11466853

[R32] Kuklenyik Z, , 2005. Measurement of 18 perfluorinated organic acids and amides in human serum using On-Line solid-phase extraction. Anal. Chem 77, 6085–6091. 16159145 10.1021/ac050671l

[R33] Kwiatkowski CF, , 2020. Scientific basis for managing PFAS as a chemical class. Environ. Sci. Technol. Lett 7, 532–543. 34307722 10.1021/acs.estlett.0c00255 PMC8297807

[R34] Lagostena L, , 2025. Persistent pollutants and the developing brain: the role of PFAS in neurodevelopmental disorders. Front. Cell. Neurosci 19–2025. 10.3389/fncel.2025.1696173 PMC12678283 41356495

[R35] Lebel C, Beaulieu C, 2011. Longitudinal development of human brain wiring continues from childhood into adulthood. J. Neurosci 31, 10937–10947. 21795544 10.1523/JNEUROSCI.5302-10.2011 PMC6623097

[R36] Liao C. y., , 2008. Acute enhancement of synaptic transmission and chronic inhibition of synaptogenesis induced by perfluorooctane sulfonate through mediation of voltage-dependent calcium channel. Environ. Sci. Technol 42, 5335–5341. 18754390 10.1021/es800018k

[R37] Liu SH, , 2024. The U.S. PFAS exposure burden calculator for 2017–2018: application to the HOME study, with comparison of epidemiological findings from NHANES. Neurotoxicol. Teratol 102, 107321. 38224844 10.1016/j.ntt.2024.107321 PMC11249202

[R38] Ma D, , 2022. A critical review on transplacental transfer of Per- and polyfluoroalkyl substances: prenatal exposure levels, characteristics, and mechanisms. Environ. Sci. Technol 56, 6014–6026. 34142548 10.1021/acs.est.1c01057

[R39] Midasch O, , 2007. Transplacental exposure of neonates to perfluorooctanesulfonate and perfluorooctanoate: a pilot study. Int. Arch. Occup. Environ. Health 80, 643–648. 17219182 10.1007/s00420-006-0165-9

[R40] Nakua H, , 2025. Investigating cross-sectional and longitudinal relationships between brain structure and distinct dimensions of externalizing psychopathology in the ABCD sample. Neuropsychopharmacology 50, 499–506. 39384894 10.1038/s41386-024-02000-3 PMC11735780

[R41] Ostby Y, , 2009. Heterogeneity in subcortical brain development: a structural magnetic resonance imaging study of brain maturation from 8 to 30 years. J. Neurosci 29, 11772–11782. 19776264 10.1523/JNEUROSCI.1242-09.2009 PMC6666647

[R42] Owens MM, , 2021. Multimethod investigation of the neurobiological basis of ADHD symptomatology in children aged 9–10: baseline data from the ABCD study. Transl. Psychiatry 11, 64. 33462190 10.1038/s41398-020-01192-8 PMC7813832

[R43] Pierozan P, Karlsson O, 2021. Differential susceptibility of rat primary neurons and neural stem cells to PFOS and PFOA toxicity. Toxicol. Lett 349, 61–68. 34126183 10.1016/j.toxlet.2021.06.004

[R44] Pinney SM, , 2014. Serum biomarkers of polyfluoroalkyl compound exposure in young girls in greater Cincinnati and the San Francisco Bay area, USA. Environ. Pollut 184, 327–334. 24095703 10.1016/j.envpol.2013.09.008 PMC3846284

[R45] Reimann GE, , 2024. Gray matter volume associations in youth with ADHD features of inattention and hyperactivity/impulsivity. Hum. Brain Mapp 45, e26589. 38530121 10.1002/hbm.26589 PMC10964792

[R46] Rosch KS, , 2024. Shared and distinct alterations in brain morphology in children with ADHD and obesity: reduced cortical surface area in ADHD and thickness in overweight/obesity. J. Psychiatr. Res 180, 103–112. 39388790 10.1016/j.jpsychires.2024.10.002 PMC11613793

[R47] Samea F, , 2019. Brain alterations in children/adolescents with ADHD revisited: a neuroimaging meta-analysis of 96 structural and functional studies. Neurosci. Biobehav. Rev 100, 1–8. 30790635 10.1016/j.neubiorev.2019.02.011 PMC7966818

[R48] Sarabin E, , 2023. The relationship between cortical thickness and executive function measures in children with and without ADHD. J. Atten. Disord 27, 1263–1271. 37183911 10.1177/10870547231174036 PMC10466945

[R49] Sharp TH, , 2023. The subcortical correlates of autistic traits in school-age children: a population-based neuroimaging study. Mol. Autism 14, 6. 36765403 10.1186/s13229-023-00538-5 PMC9921646

[R50] Shaw P, , 2008. Neurodevelopmental trajectories of the human cerebral cortex. J. Neurosci 28, 3586–3594. 18385317 10.1523/JNEUROSCI.5309-07.2008 PMC6671079

[R51] Smith SM, Nichols TE, 2009. Threshold-free cluster enhancement: addressing problems of smoothing, threshold dependence and localisation in cluster inference. Neuroimage 44, 83–98. 18501637 10.1016/j.neuroimage.2008.03.061

[R52] Starnes HM, , 2022. A critical review and meta-analysis of impacts of Per- and polyfluorinated substances on the brain and behavior. Front. Toxicol 4, 881584. 35480070 10.3389/ftox.2022.881584 PMC9035516

[R53] Sultan H, , 2023. Dietary per- and polyfluoroalkyl substance (PFAS) exposure in adolescents: the HOME study. Environ. Res 231, 115953. 37142081 10.1016/j.envres.2023.115953 PMC10330479

[R54] Suzuki M, , 2025. Number of carbons is a critical parameter for accumulation of Per- and polyfluoroalkyl substances in the human brain. Environ. Sci. Technol 59, 3366–3375. 39927984 10.1021/acs.est.4c09458

[R55] Tamnes CK, , 2010. Brain maturation in adolescence and young adulthood: regional age-related changes in cortical thickness and white matter volume and microstructure. Cerebr. Cortex 20, 534–548. 10.1093/cercor/bhp118 19520764

[R56] VanNoy BN, , 2018. Breastfeeding as a predictor of serum concentrations of Per- and polyfluorinated alkyl substances in reproductive-aged women and young children: a rapid systematic review. Curr. Environ. Health Rep 5, 213–224. 29737463 10.1007/s40572-018-0194-z

[R57] Viner RM, , 2015. Life course epidemiology: recognising the importance of adolescence. J. Epidemiol. Community Health 69, 719–720. 25646208 10.1136/jech-2014-205300 PMC4515995

[R58] Vuong AM, , 2021a. Prenatal exposure to per- and polyfluoroalkyl substances (PFAS) and neurobehavior in US children through 8 years of age: the HOME study. Environ. Res 195, 110825. 33545124 10.1016/j.envres.2021.110825 PMC7987860

[R59] Vuong AM, , 2021b. Childhood exposure to per- and polyfluoroalkyl substances (PFAS) and neurobehavioral domains in children at age 8 years. Neurotoxicol. Teratol 88, 107022. 34438039 10.1016/j.ntt.2021.107022 PMC8578387

[R60] Wang J, , 2018. Penetration of PFASs across the blood cerebrospinal fluid barrier and its determinants in humans. Environ. Sci. Technol 52, 13553–13561. 30362723 10.1021/acs.est.8b04550

[R61] Wang SM, , 2025. White matter microstructural integrity mediates associations between prenatal endocrine-disrupting chemicals exposure and intelligence in adolescents. Neuroimage, Clin 45, 103758. 39983551 10.1016/j.nicl.2025.103758 PMC11889738

[R62] Wu X, , 2023. Morphometric dis-similarity between cortical and subcortical areas underlies cognitive function and psychiatric symptomatology: a preadolescence study from ABCD. Mol. Psychiatr 28, 1146–1158. 10.1038/s41380-022-01896-x 36473996

[R63] Zhang Z, , 2022. Biodegradation of per- and polyfluoroalkyl substances (PFAS): a review. Bioresour. Technol 344, 126223. 34756980 10.1016/j.biortech.2021.126223

