# Supplementary material for: Serum PFAS concentrations and neuromorphometry in adolescents: The HOME Study
Source: Environ Res. Author manuscript; Available in PMC 2026 May 13. (PMC13162322; doi:10.1016/j.envres.2026.124338)
Supplement: Supplementary Material-Tables [file NIHMS2170022-supplement-Supplementary_Material-Tables.docx]

**Supplemental Table 1.** Secondary analysis of associations of log_2_-transformed serum PFAS concentrations with whole-brain morphometric measurements with concurrent timepoint covariates. Gestational age analyses adjusted for maternal IQ, household income at baseline, maternal race, pre-pregnancy BMI, primipara, adolescent sex and total intracranial volume. Age 12 analyses adjusted for maternal IQ, household income at 12y, adolescent race, adolescent BMI, adolescent sex and total intracranial volume. Cohen’s effect sizes (f^2^), p-values, Beta coefficients (β) and 95% confidence intervals are presented. Volumes of gray matter, white matter and cerebral spinal fluid (CSF) units are in cubic centimeters. Cortical thickness units are in millimeters. PFAS abbreviations: perfluorooctanoic acid (PFOA), perfluorooctanesulfonic acid (PFOS), perfluorononanoic acid (PFNA), perfluorohexane sulfonic acid (PFHxS), and perfluorodecanoic acid (PFDA).

|  |  | | |  | | **Gray Matter** | |  | | | **White Matter** | | |  | | **CSF** | |  | | | **Average Cortical Thickness** | |  |
| --- | --- | --- | --- | --- | --- | --- | --- | --- | --- | --- | --- | --- | --- | --- | --- | --- | --- | --- | --- | --- | --- | --- | --- |
| **Exposure Window** | **PFAS** | N | *f^2^* | | β (95% C.I.) | | p-value | | *f^2^* | β (95% C.I.) | | p-value | *f^2^* | | β (95% C.I.) | | p-value | | *f^2^* | β (95% C.I.) | | p-value |  |
| Gestational | PFOA | 155 | 0 | | 0.516 (-4.542, 5.574) | | 0.841 | | 0 | -1.678 (-6.314, 2.957) | | 0.475 | 0 | | 1.162 (-3.832, 6.157) | | 0.646 | | 0 | 0.002 (-0.018, 0.023) | | 0.818 |  |
|  | PFOS | 155 | 0 | | 0.142 (-4.402, 4.687) | | 0.951 | | 0 | 0.774 (-3.396, 4.943) | | 0.714 | 0 | | -0.916 (-5.404, 3.572) | | 0.687 | | 0 | -0.004 (-0.022, 0.014) | | 0.678 |  |
|  | PFNA | 155 | 0.01 | | 3.664 (-2.111, 9.438) | | 0.212 | | 0.01 | -3.692 (-8.987, 1.603) | | 0.17 | 0 | | 0.028 (-5.708, 5.764) | | 0.992 | | 0.01 | 0.014 (-0.010, 0.037) | | 0.249 |  |
|  | PFHXS | 155 | 0 | | -1.056 (-4.562, 2.450) | | 0.553 | | 0.02 | 2.504 (-0.693, 5.700) | | 0.124 | 0 | | -1.447 (-4.908, 2.013) | | 0.41 | | 0 | -0.004 (-0.018, 0.010) | | 0.561 |  |
|  | PFDA | 155 | 0.02 | | 3.807 (-0.769, 8.383) | | 0.102 | | 0.02 | -3.394 (-7.597, 0.808) | | 0.113 | 0 | | -0.413 (-4.975, 4.149) | | 0.858 | | 0.02 | 0.016 (-0.003, 0.034) | | 0.096 |  |
| Age 12 | PFOA | 154 | ***0.04*** | | ***9.060 (1.666, 16.454)*** | | ***0.017*** | | ***0.04*** | ***-8.327 (-15.234, -1.420)*** | | ***0.018*** | 0 | | -0.733 (-7.629, 6.163) | | 0.834 | | ***0.03*** | ***0.032 (0.001, 0.062)*** | | ***0.042*** |  |
|  | PFOS | 154 | 0.02 | | 4.183 (-1.101, 9.466) | | 0.12 | | ***0.03*** | ***-5.051 (-9.955, -0.1460)*** | | ***0.044*** | 0 | | 0.868 (-4.003, 5.739) | | 0.725 | | ***0.03*** | ***0.023 (0.001, 0.045)*** | | ***0.037*** |  |
|  | PFNA | 154 | ***0.03*** | | ***4.934 (-0.128, 9.995)*** | | ***0.056*** | | 0.01 | -2.240 (-7.011, 2.2532) | | 0.355 | 0.01 | | -2.694 (-7.361, 1.973) | | 0.256 | | 0.01 | 0.015 (-0.006, 0.036) | | 0.168 |  |
|  | PFHXS | 154 | 0 | | 0.647 (-2.728, 4.022) | | 0.705 | | 0 | -0.594 (-3.744, 2.557) | | 0.71 | 0 | | -0.054 (-3.142, 3.035) | | 0.973 | | 0 | 0.001 (-0.013, 0.015) | | 0.852 |  |
|  | PFDA | 154 | 0.01 | | 4.516 (-1.800, 10.832) | | 0.16 | | 0.01 | -3.347 (-9.528, 2.565) | | 0.265 | 0 | | -1.169 (-6.982, 4.644) | | 0.692 | | 0 | 0.001 (-0.026, 0.027) | | 0.965 |  |

**Supplemental Table 2.** Sensitivity analysis removing maternal full scale intelligence quotient in the associations of log_2_-transformed serum PFAS concentrations with whole-brain morphometric measurements, adjusting for sex, race, household income, maternal pre-pregnancy BMI, primipara and total intracranial volume for each exposure window. Cohen’s effect sizes (f^2^), p-values, Beta coefficients (β) and 95% confidence intervals are presented. Volumes of gray matter, white matter and cerebral spinal fluid (CSF) units are in cubic centimeters. Cortical thickness units are in millimeters. PFAS abbreviations: perfluorooctanoic acid (PFOA), perfluorooctanesulfonic acid (PFOS), perfluorononanoic acid (PFNA), perfluorohexane sulfonic acid (PFHxS), and perfluorodecanoic acid (PFDA).

|  |  | | |  | | **Gray Matter** | |  | | | **White Matter** | | |  | | **CSF** | |  | | | **Average Cortical Thickness** | |  |
| --- | --- | --- | --- | --- | --- | --- | --- | --- | --- | --- | --- | --- | --- | --- | --- | --- | --- | --- | --- | --- | --- | --- | --- |
| **Exposure Window** | **PFAS** | N | *f^2^* | | β (95% C.I.) | | p-value | | *f^2^* | β (95% C.I.) | | p-value | *f^2^* | | β (95% C.I.) | | p-value | | *f^2^* | β (95% C.I.) | | p-value |  |
| Gestational | PFOA | 155 | 0 | | 0.331 (-4.595, 5.258) | | 0.894 | | 0 | -0.958 (-5.470, 3.554) | | 0.675 | 0 | | 0.627 (-4.240, 5.494) | | 0.800 | | 0 | 0.001 (-0.019, 0.021) | | 0.938 |  |
|  | PFOS | 155 | 0 | | 0.555 (-3.941, 5.050) | | 0.808 | | 0 | 0.784 (-3.335, 4.902) | | 0.707 | 0 | | -1.339 (-5.776, 3.099) | | 0.552 | | 0 | -0.003 (-0.021, 0.015) | | 0.770 |  |
|  | PFNA | 155 | 0.01 | | 4.051 (-1.735, 9.836) | | 0.169 | | 0.01 | -3.795 (-9.096, 1.505) | | 0.159 | 0 | | -0.256 (-6.009, 5.498) | | 0.930 | | 0.01 | 0.015 (-0.008, 0.038) | | 0.208 |  |
|  | PFHXS | 155 | 0 | | -1.350 (-4.842, 2.142) | | 0.446 | | 0.02 | 3.064 (-0.103, 6.231) | | 0.058 | 0.01 | | -1.714 (-5.160, 1.732) | | 0.327 | | 0 | -0.004 (-0.019, 0.010) | | 0.528 |  |
|  | PFDA | 155 | 0.02 | | 4.248 (-0.345, 8.841) | | 0.070 | | 0.02 | -3.608 (-7.824, 0.608) | | 0.093 | 0 | | -0.640 (-5.228, 3.948) | | 0.783 | | 0.02 | 0.017 (-0.001, 0.036) | | 0.067 |  |
| Age 12 | PFOA | 153 | ***0.06*** | | ***10.615 (3.250, 17.980)*** | | ***0.005*** | | ***0.04*** | ***-8.249 (-15.127, -1.370)*** | | ***0.019*** | 0 | | -2.367 (-9.238, 4.504) | | 0.497 | | ***0.03*** | ***0.032 (0.002, 0.062)*** | | ***0.039*** |  |
|  | PFOS | 153 | 0.02 | | 4.941 (-0.331, 10.212) | | 0.066 | | ***0.03*** | ***-5.030 (-9.901, -0.159)*** | | ***0.043*** | 0 | | 0.089 (-4.761, 4.939) | | 0.971 | | ***0.03*** | ***0.023 (0.002, 0.044)*** | | ***0.035*** |  |
|  | PFNA | 153 | ***0.04*** | | ***6.395 (1.360, 11.430)*** | | ***0.013*** | | 0.01 | -2.876 (-7.617, 1.865) | | 0.232 | 0.02 | | -3.519 (-8.160, 1.122) | | 0.136 | | 0.02 | 0.016 (-0.005, 0.037) | | 0.133 |  |
|  | PFHXS | 153 | 0 | | 0.427 (-3.068, 3.922) | | 0.809 | | 0 | -0.453 (-3.690, 2.785) | | 0.783 | 0 | | 0.025 (-3.153, 3.204) | | 0.987 | | 0 | 0.002 (-0.012, 0.016) | | 0.798 |  |
|  |  |  |  | |  | |  | |  |  | |  |  | |  | |  | |  |  | |  |  |

**Supplemental Table 3**. Results for Sex Interaction for Global Analyses of the Primary Models. PFAS abbreviations: perfluorooctanoic acid (PFOA), perfluorooctanesulfonic acid (PFOS), perfluorononanoic acid (PFNA), perfluorohexane sulfonic acid (PFHxS), and perfluorodecanoic acid (PFDA).

| Exposure Window | PFAS | Metric | N | *f^2^* | β (95% C.I.) | p-value |
| --- | --- | --- | --- | --- | --- | --- |
| Age 12 yr | PFOA | Gray Matter | 153 | 0.02 | -9.319 (-21.601, 2.964) | 0.136 |
|  | PFOA | White Matter | 153 | 0 | -1.104 (-12.564, 10.356) | 0.849 |
|  | PFOA | Avg. Cortical Thickness | 153 | 0 | -0.014 (-0.065, 0.037) | 0.585 |
|  | PFOS | White Matter | 153 | 0.01 | -5.815 (-14.850, 3.220) | 0.205 |
|  | PFOS | Avg. Cortical Thickness | 153 | 0 | 0.012 (-0.028, 0.052) | 0.552 |
|  | PFNA | Gray Matter | 153 | 0.01 | -4.905 (-14.077, 4.266) | 0.292 |
