# Supplementary material for: Serum PFAS concentrations and neuromorphometry in adolescents: The HOME Study
Source: Environ Res. Author manuscript; Available in PMC 2026 May 13. (PMC13162322; doi:10.1016/j.envres.2026.124338)
Supplement: Supplementary Material-Figures [file NIHMS2170022-supplement-Supplementary_Material-Figures.docx]

**Supplemental Figures**

**Supplemental Figure 1.** Scatterplot illustrating the association for log_2_PFOA concentration at age 12y and concurrent total gray matter volume in cubic centimeters (cm^3^) for our primary model adjusting for sex, race, household income, maternal IQ, maternal pre-pregnancy BMI, primipara and total intracranial volume.


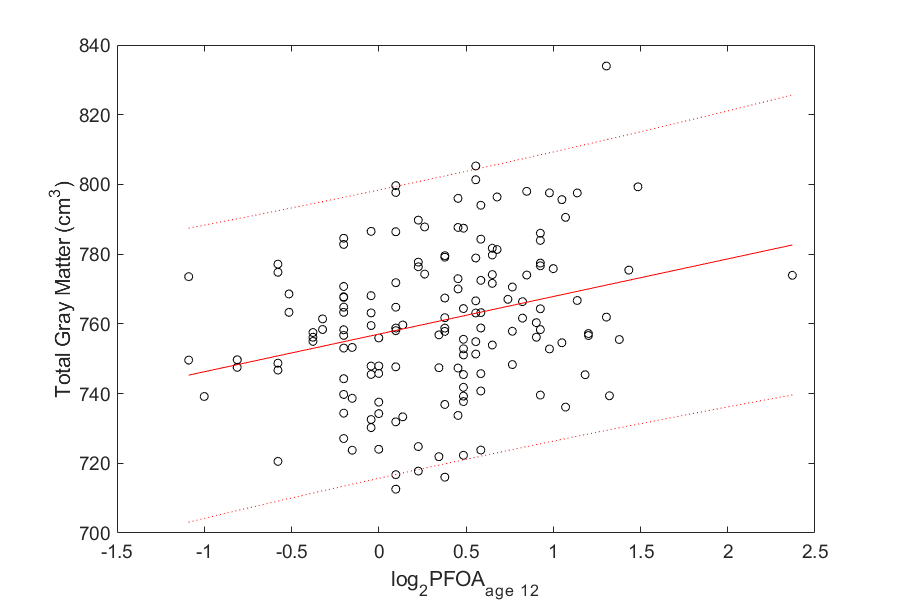


**Supplemental Figure 2**. Scatterplot illustrating the association for log_2_PFNA concentration at age 12y and concurrent total gray matter volume in cubic centimeters (cm^3^) model adjusting for sex, race, household income, maternal IQ, maternal pre-pregnancy BMI, primipara and total intracranial volume.


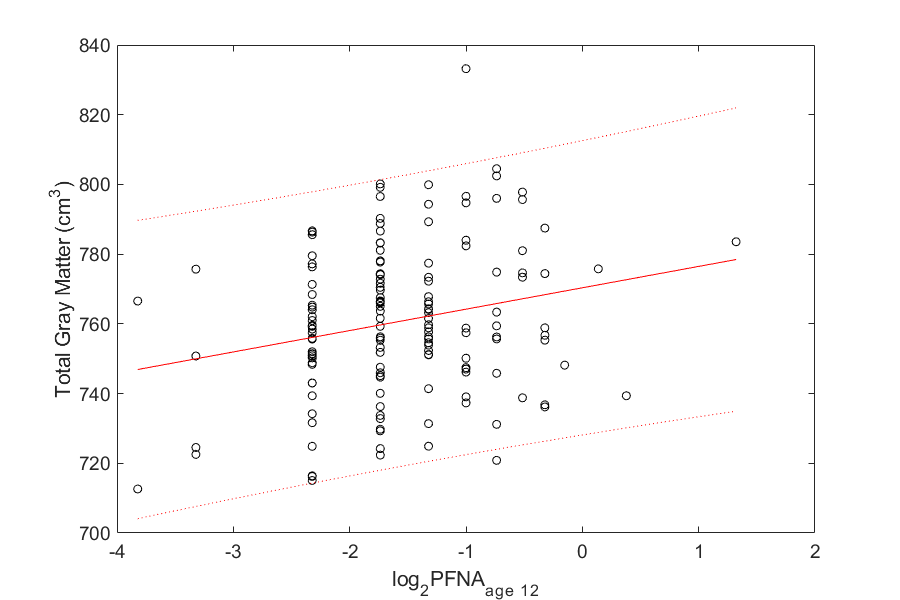


**Supplemental Figure 3.** Scatterplot illustrating the association for log_2_PFOA concentration at age 12y and concurrent total white matter volume in cubic centimeters (cm^3^) model adjusting for sex, race, household income, maternal IQ, maternal pre-pregnancy BMI, primipara and total intracranial volume.


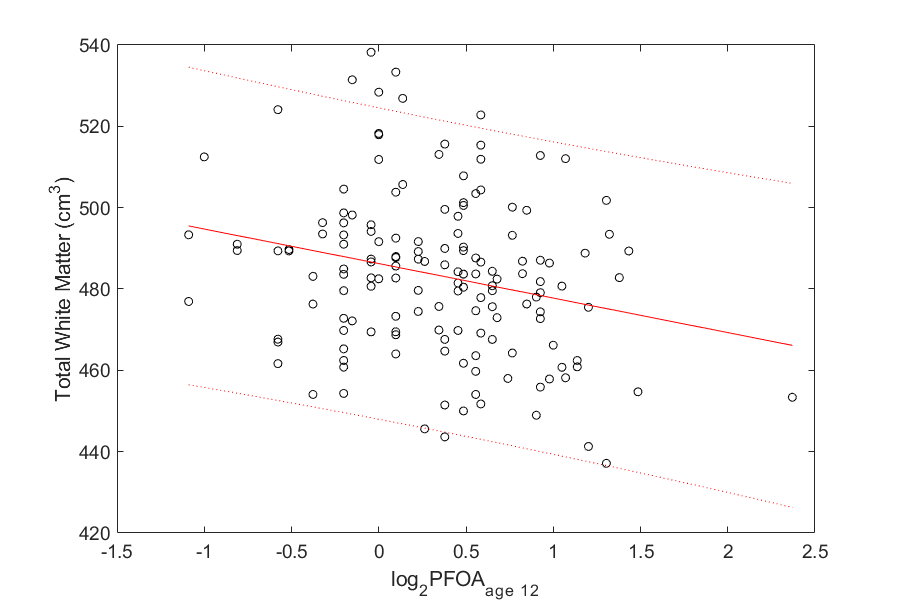


**Supplemental Figure 4.** Scatterplot illustrating the association for log_2_PFOS concentration at age 12y and concurrent total white matter volume in cubic centimeters (cm^3^) model adjusting for sex, race, household income, maternal IQ, maternal pre-pregnancy BMI, primipara and total intracranial volume.


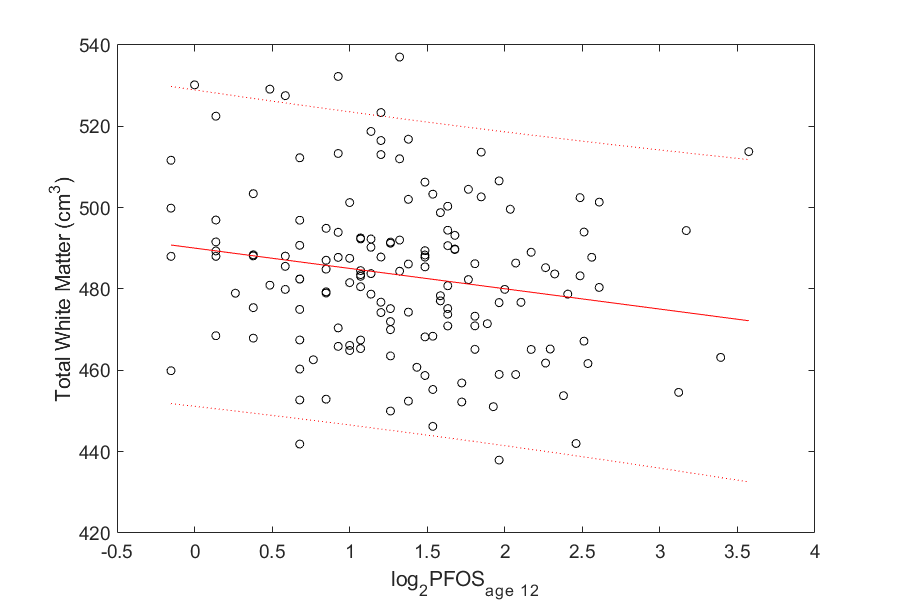


**Supplemental Figure 5.** Scatterplot illustrating the association for log_2_PFOA concentration at age 12y and concurrent whole brain average cortical thickness in millimeters (mm) model adjusting for sex, race, household income, maternal IQ, maternal pre-pregnancy BMI, primipara and total intracranial volume.


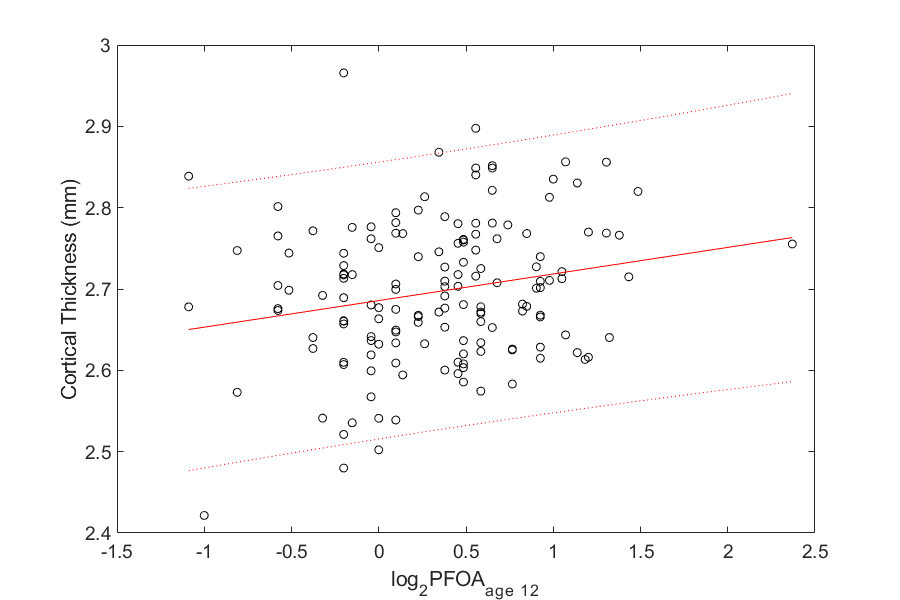


**Supplemental Figure 6**. Scatterplot illustrating the association for log_2_PFOS concentration at age 12y and concurrent whole brain average cortical thickness in millimeters (mm) model adjusting for sex, race, household income, maternal IQ, maternal pre-pregnancy BMI, primipara and total intracranial volume.


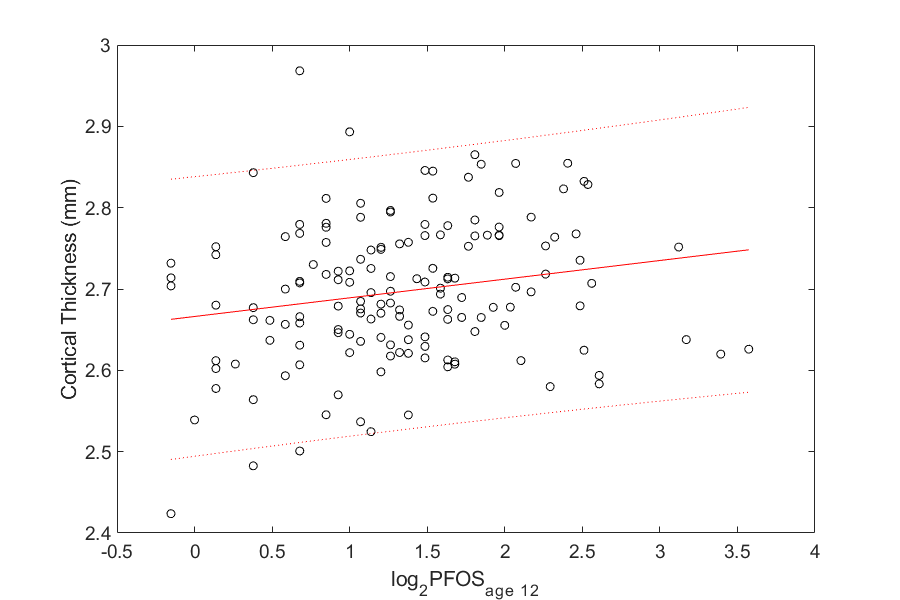


**Supplemental Figure 7** Sensitivity analysis results upon removing maternal full scale intelligence quotient in the associations of log_2_-transformed serum perfluorooctanoic acid (PFOA) concentrations with whole-brain morphometric measurements, adjusting for sex, race, household income, maternal IQ, maternal pre-pregnancy BMI, primipara and total intracranial volume. The figure shows a T1-weighted imaging template illustrating beta-coefficients of spatially localized regions demonstrating the associations identified from the VBM analyses and PFOA concentrations at the age 12 study visit.


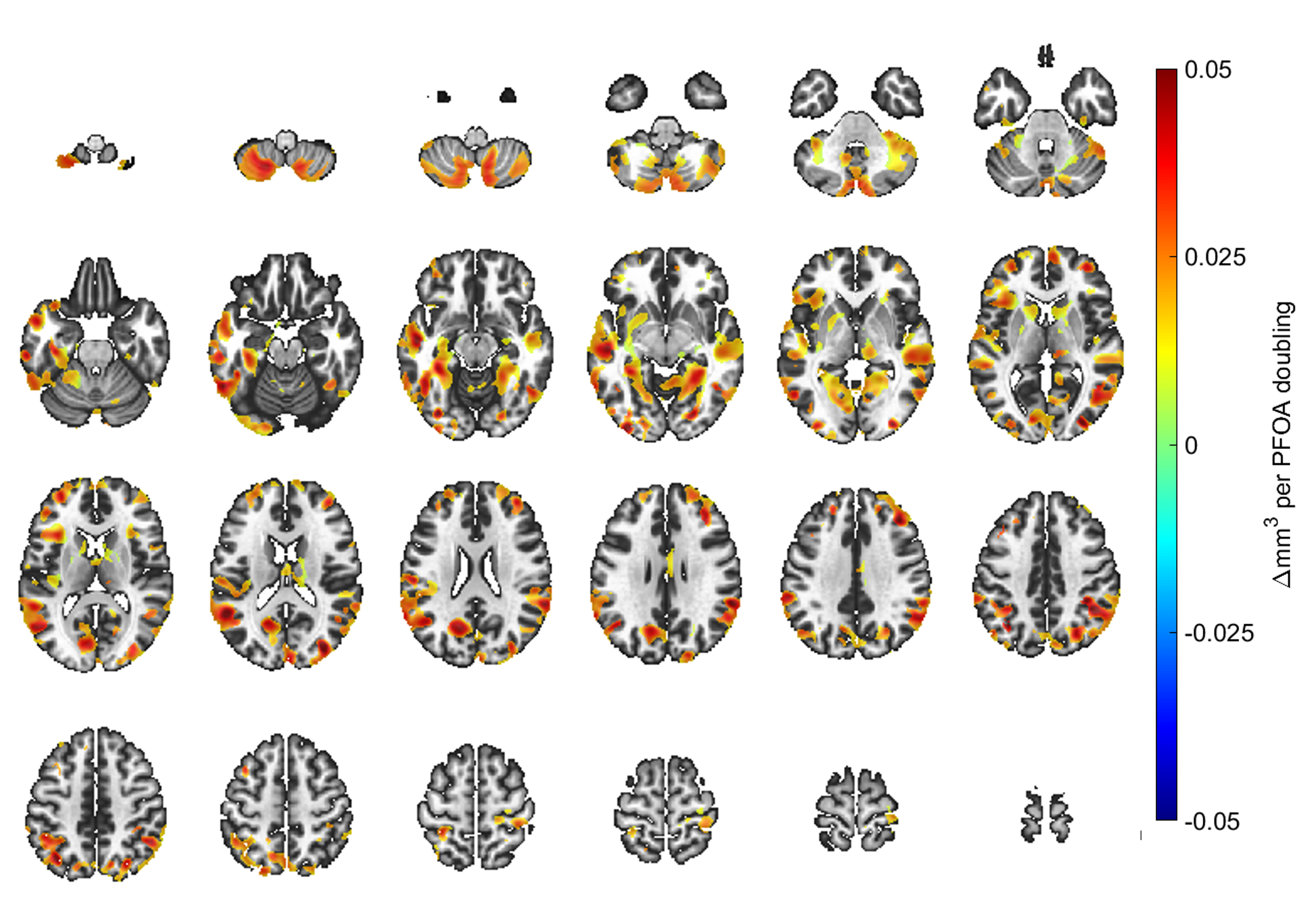


**Supplemental Figure 8.** Sensitivity analysis results upon removing maternal full scale intelligence quotient in the associations of log_2_-transformed serum perfluorononanoic acid (PFNA) concentrations with whole-brain morphometric measurements, adjusting for sex, race, household income, maternal IQ, maternal pre-pregnancy BMI, primipara and total intracranial volume. The figure shows a T1-weighted imaging template illustrating beta-coefficients of spatially localized regions demonstrating the associations identified from the VBM analyses and PFNA concentrations at the age 12 study visit.


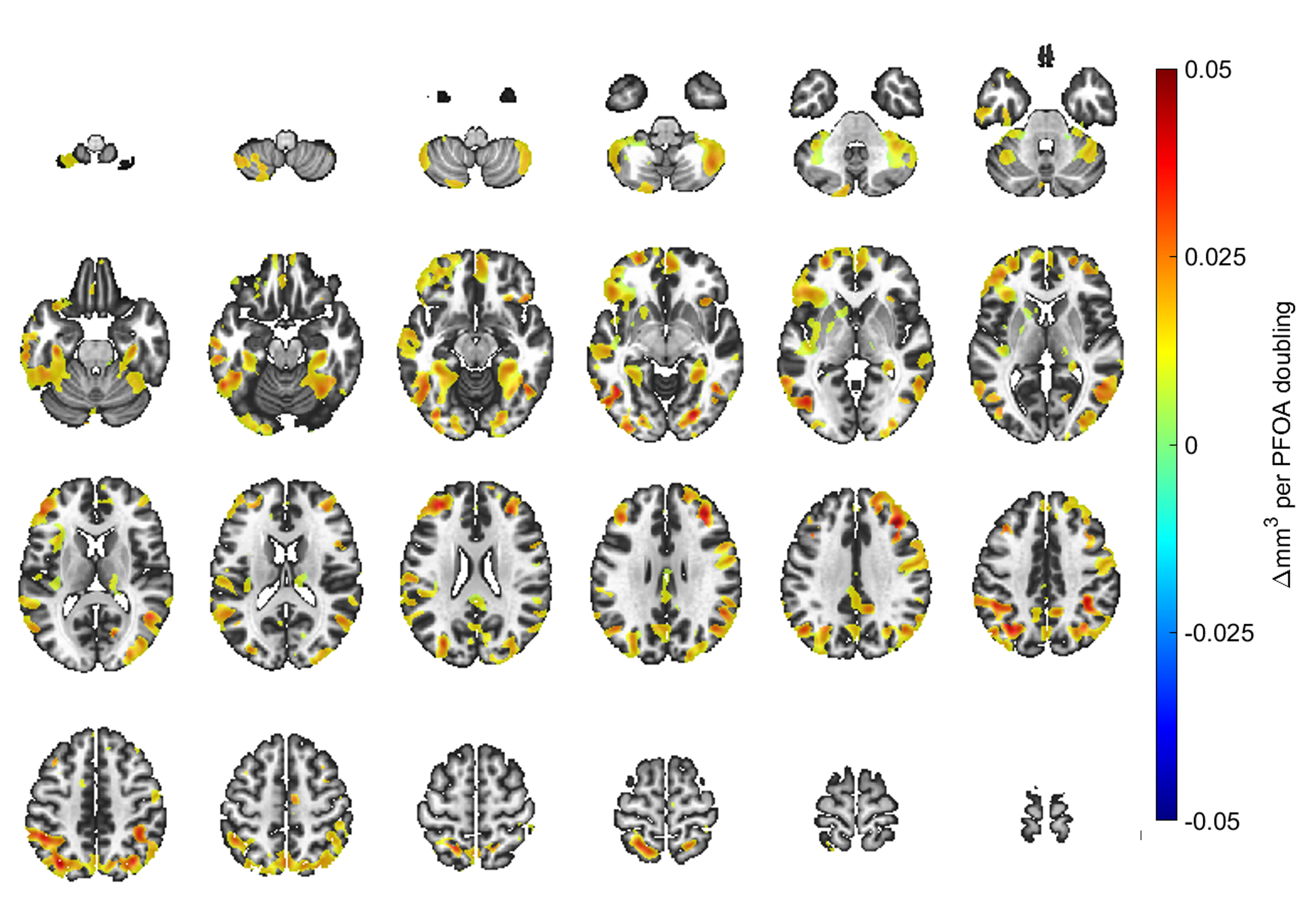


**Supplemental Figure 10.** Secondary Model analysis with maternal IQ, household income at 12y, child race, child BMI, child sex as covariates. Illustrative brain renderings featuring beta-coefficients for regions where cortical thickness was associated with concurrent concentrations of perfluorooctanoic acid (PFOA) at the age 12 study visit. Top row from left to right represents the view of the left hemisphere, the superior view, and view of the right hemisphere. Bottom row from left to right represents the midline perspective of the left hemisphere, the inferior view and the midline view of the right hemisphere. As noted by the color scale, the change in mm corresponds to a doubling of the concurrent PFOA concentrations. Regions shown in the darkest red correspond to a 0.1 mm increase with a doubling of the concurrent PFOA concentration.


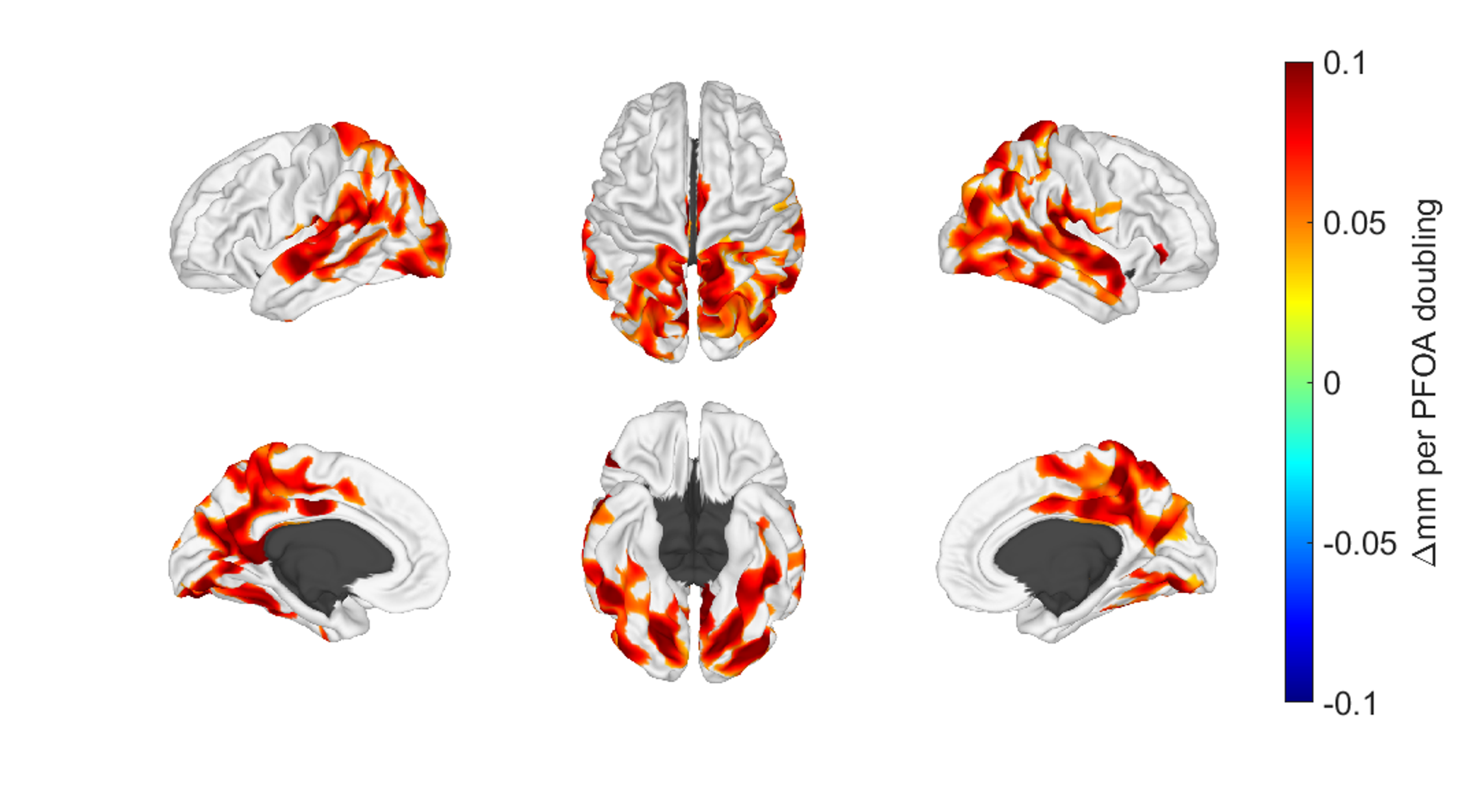


**Supplemental Figure 11**. Secondary Model analysis with maternal IQ, household income at 12y, child race, child BMI, child sex as covariates. Illustrative brain renderings featuring beta-coefficients for regions where cortical thickness was associated with concurrent perfluorooctanesulfonic acid (PFOS) concentrations at the age 12 study visit. Top row from left to right represents the view of the left hemisphere, the superior view, and view of the right hemisphere. Bottom row from left to right represents the midline perspective of the left hemisphere, the inferior view and the midline view of the right hemisphere. As noted by the color scale, the change in mm corresponds to a doubling of the concurrent PFOS concentrations. Regions shown in the darkest red correspond to a 0.1 mm increase with a doubling of the concurrent PFOS concentration.


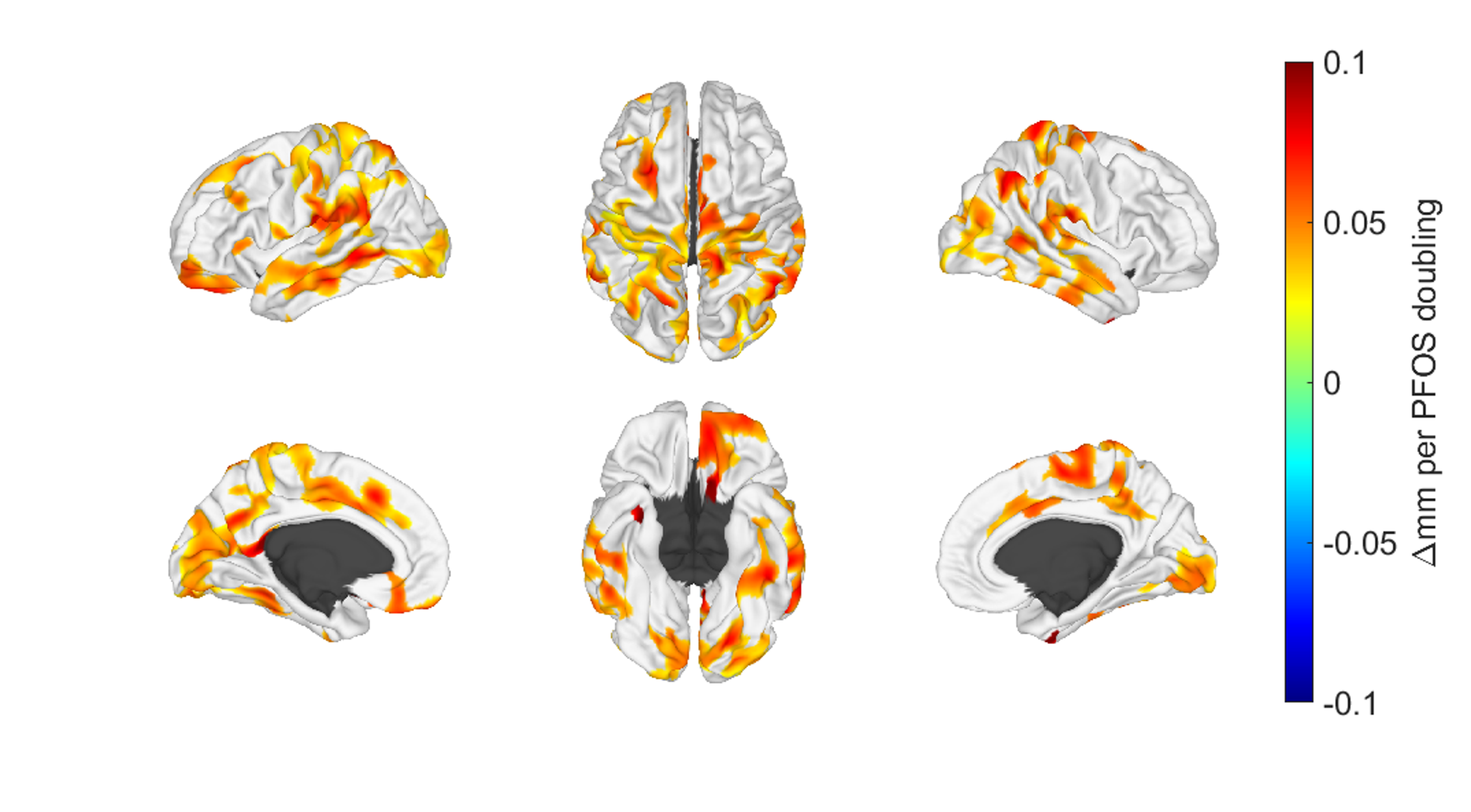


**Supplemental Figure 12.** Sensitivity analysis results upon removing maternal full scale intelligence quotient in the associations of log_2_-transformed serum perfluorooctanoic acid (PFOA) concentrations with whole-brain morphometric measurements, adjusting for sex, race, household income, maternal IQ, maternal pre-pregnancy BMI, primipara and total intracranial volume. The figure shows Illustrative brain renderings featuring beta-coefficients for regions where cortical thickness was associated with concurrent concentrations of PFOA at the age 12 study visit.

**
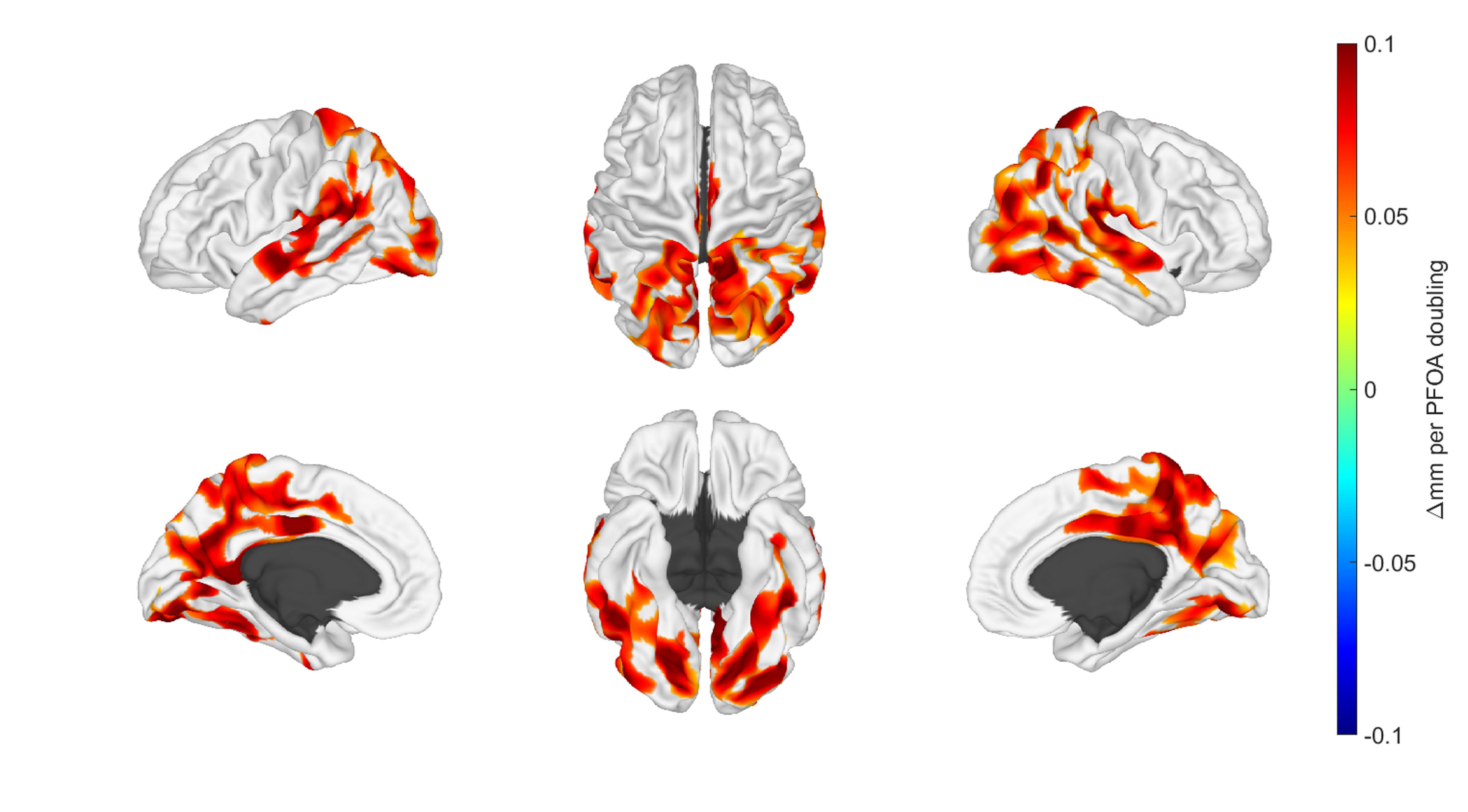
**

**Supplemental Figure 13.** Sensitivity analysis results upon removing maternal full scale intelligence quotient in the associations of log_2_-transformed serum perfluorononanoic acid (PFNA) concentrations with whole-brain morphometric measurements, adjusting for sex, race, household income, maternal IQ, maternal pre-pregnancy BMI, primipara and total intracranial volume. The figure shows Illustrative brain renderings featuring beta-coefficients for regions where cortical thickness was associated with concurrent concentrations of PFOS at the age 12 study visit

**
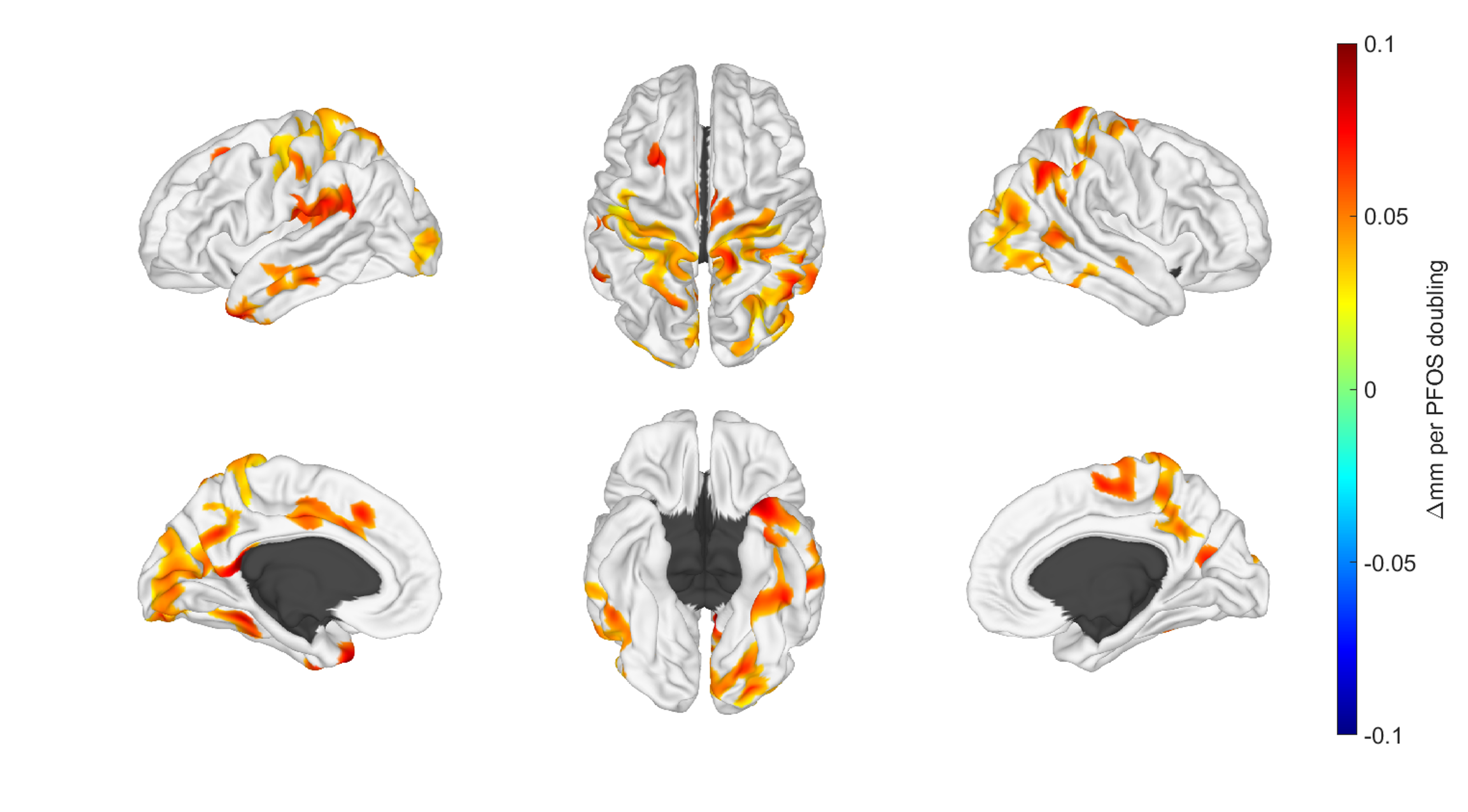
**

**Supplemental Figure 14.** Un-thresholded effect size maps as brain renderings featuring beta-coefficients for regions where cortical thickness was associated with concurrent concentrations of perfluorooctanoic acid (PFOA) at the age 12 study visit were explored for convergence with other published results.

**
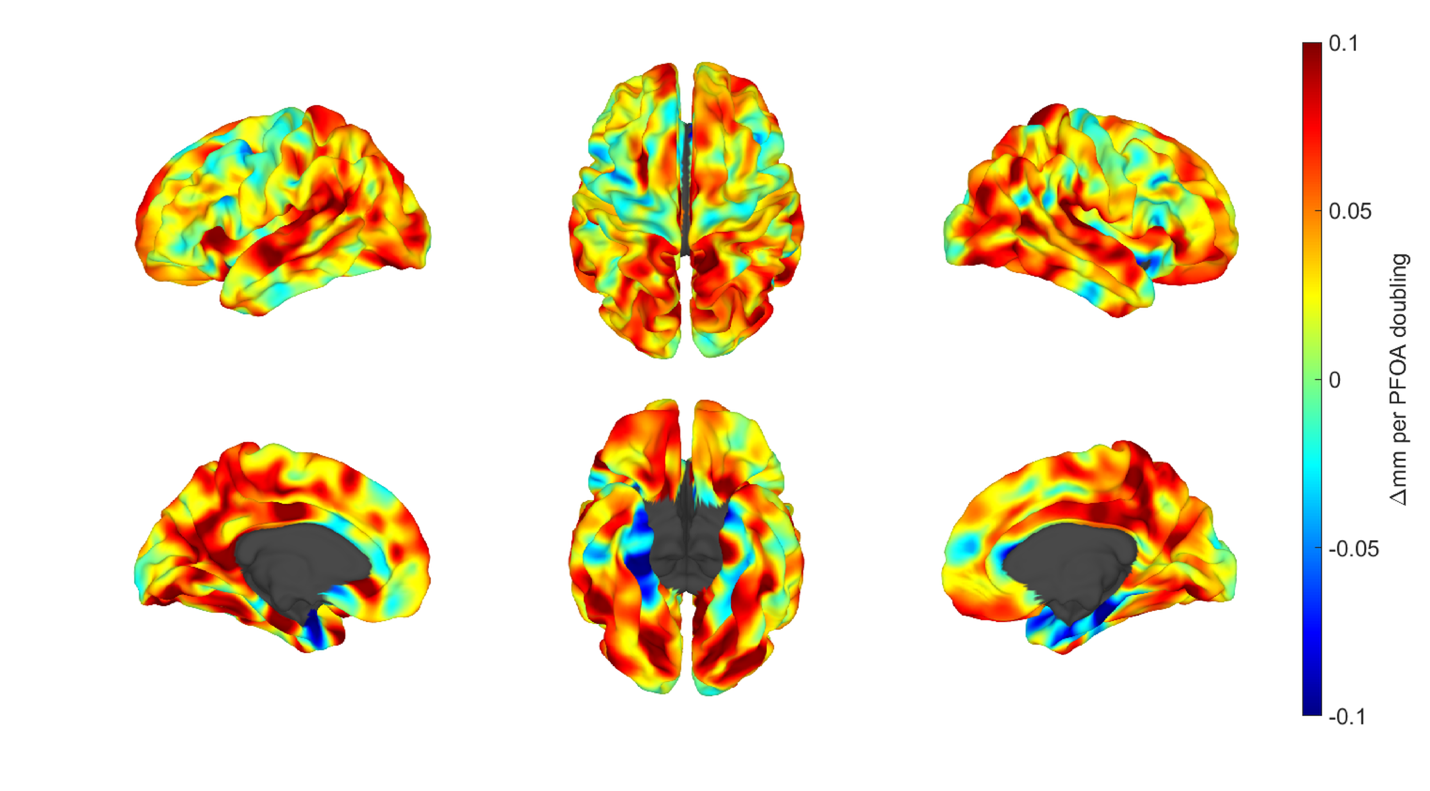
**

**Supplemental Figure 15.** Un-thresholded effect size maps as brain renderings featuring beta-coefficients for regions where cortical thickness was associated with concurrent perfluorooctanesulfonic acid (PFOS) concentrations at age 12 study visit were explored for convergence with other published results.

**
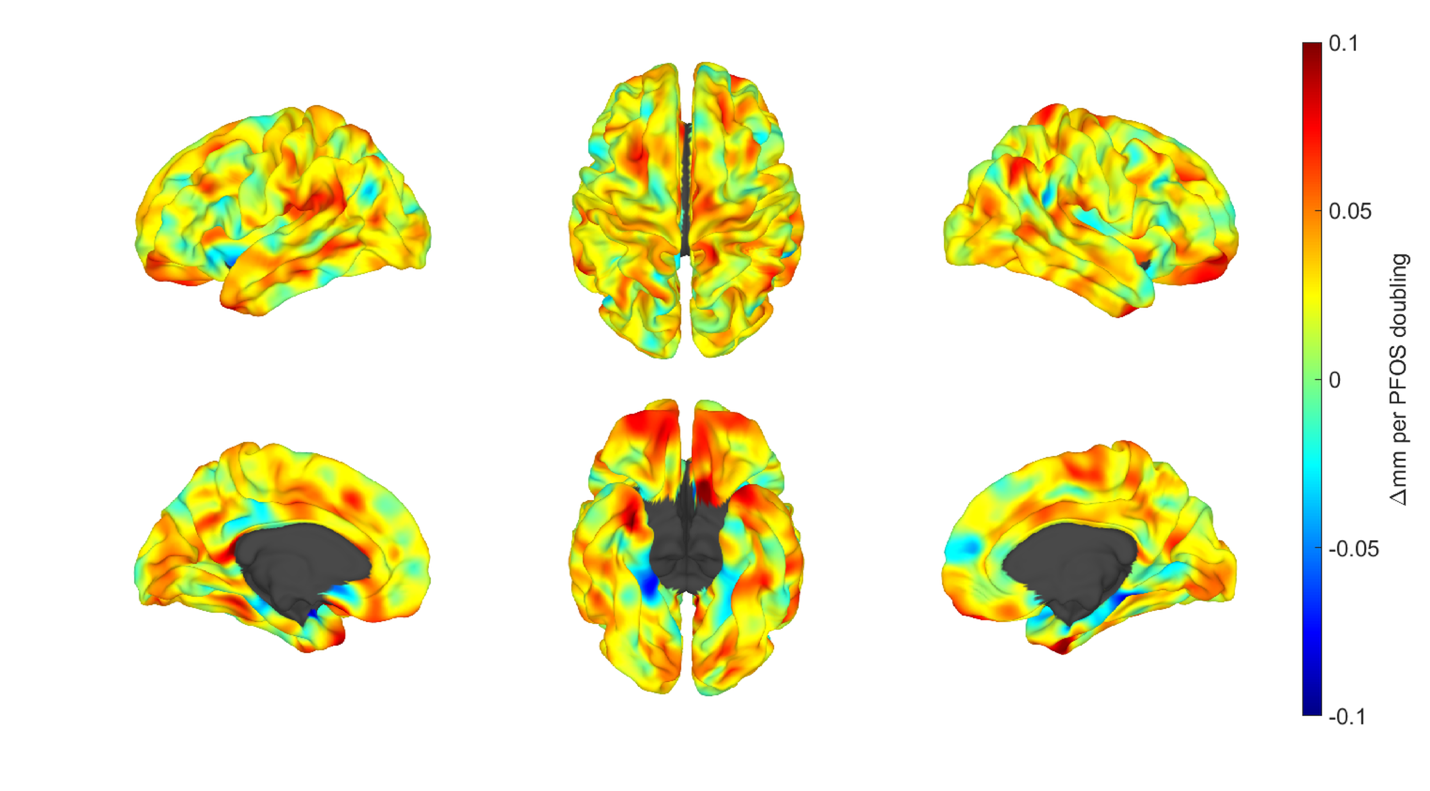
**
